# Diet, growth, and obesity development throughout childhood in the Avon Longitudinal Study of Parents and Children

**DOI:** 10.1093/nutrit/nuv054

**Published:** 2015-09-22

**Authors:** Pauline M. Emmett, Louise R. Jones

**Affiliations:** *P.M. Emmett* is with the Centre for Child and Adolescent Health, School of Social and Community Medicine, University of Bristol, Bristol, United Kingdom.; *L.R. Jones* is with the School of Social and Community Medicine, University of Bristol, Bristol, United Kingdom.

**Keywords:** ALSPAC, childhood diet, diet, energy density, fat mass, fruit and vegetables, growth, inequality, obesity, sugar

## Abstract

Publications from the Avon Longitudinal Study of Parents and Children covering diet, growth, and obesity development during childhood are reviewed. Diet was assessed by food frequency questionnaires and food records. Growth data were collected by routine measurements, and in standardized clinics, body fatness was assessed by bioelectrical impedance and DXA (dual-energy X-ray absorptiometry) scans. Diets changed dramatically during the preschool period with an increase in the intake of free (added) sugars (12.3% rising to 16.4% of energy) that remained similar until adolescence. This was due to increased intake of energy-dense, nutrient-poor foods. Two periods of rapid growth were identified; infancy and mid-childhood (ages 7–11 y) and both were associated with obesity development. Diets with high energy density were associated with increasing fat mass from mid-childhood until adolescence. Genetic and dietary factors showed independent associations with increasing adiposity. At all ages studied, there were dietary inequalities related to maternal educational attainment that may influence inequalities found in obesity development. The Avon Longitudinal Study of Parents and Children has provided valuable insights into how disparities in diet and growth may affect the development of ill health in adulthood.

## INTRODUCTION

A balanced diet in childhood is very important to ensure optimum growth and development at a time of rapid growth. Poor diet has been associated with many adult health conditions, such as coronary heart disease, diabetes, and some cancers.^1–3^ Establishing and maintaining healthy eating habits is important because habits formed in early life are likely to continue into adulthood.^4^ The Bogalusa Heart Study found that coronary atherosclerosis and essential hypertension can begin in childhood.^5^^,^^6^ Other work has shown that childhood fruit consumption may have a long-term protective effect on cancer risk in adulthood.^7^ Therefore, an understanding of how dietary habits develop during childhood and adolescence would be informative, especially for establishing critical points for intervention to prevent the development of later health problems.

Obesity is a chronic health condition that can manifest itself well before adulthood, and there has been a rapid rise in the prevalence of childhood obesity in recent years in the Western world.^8–10^ There is evidence that rapid weight gain in early childhood is predictive of the development of obesity during childhood.^11^ It may be that early childhood is important because it is when particular dietary habits and sedentary behavior patterns become established.^12^ In later childhood and adolescence, promoting a healthy lifestyle with a balance between diet and physical activity is essential to arrest obesity development.^13^ In particular, the World Health Organization has identified the energy density of diets and their fiber content as important factors for determining obesity risk.^1^

The Avon Longitudinal Study of Parents and Children (ALSPAC) has collected comprehensive dietary information and growth measures throughout childhood and has followed obesity development into adolescence. In this respect, it has a unique combination of longitudinal data collected by hands-on standardized procedures, as well as from parental or self-completed questionnaires and routine community and health service assessments. This review aims to amalgamate the publications that used ALSPAC data and covered childhood diet, growth, and obesity development. It includes all published articles that examine food and nutrient intakes throughout the childhood of the cohort and those that relate diet and growth to obesity and other markers of health. The relationship of diet, growth, and obesity development with socioeconomic background (SEB) is explored. The diet and growth data were collected from the same children at set ages ranging from preschool to mid–secondary school age. Other reviews in the supplement of which this article is a part cover the pregnancy diet and the dietary patterns in the ALSPAC. A review of the ALSPAC’s contribution to the understanding of infancy diet and growth is published elsewhere.^14^

## LITERATURE AND STUDY METHODS

### Literature

This narrative review includes all articles using data from the ALSPAC that deal with dietary intake, growth, and obesity development between the ages of 1.5 and 15 years. The included articles are listed in Table 1. Of the 57 articles identified^15^*^–^*^72^ 4 used dietary questionnaire data, 28 used food record data, 8 assessed growth, 32 assessed obesity by body mass index (BMI) or fat mass, and 8 focused on diet and growth in relation to other health outcomes.

**Table 1:** Characteristics of the ALSPAC articles included in the present review

Reference	Dietary method (sample)	Age at assessment	Type of analysis	Focus of paper
Cowin et al. (2000)^15^	FR (ss)	1.5 y	XS	Diet description and adequacy
Emmett et al. (2002)^16^	FR (ss)	1.5 y and 3.5 y	XS and L	Diet description and adequacy
				Change in foods and nutrients
Glynn et al. (2005)^17^	FR (ss)	7 y	XS	Diet description and adequacy
				Sex differences
Cribb et al. (2011)^18^	FR (AA)	10 y	XS	Diet description and adequacy
				SEB differences
				Misreporting of energy intake (EI)
Anderson et al. (2013)^19^	FR and FFQ (AA)	All ages 3–13 y	XS and L	Trajectory of EI by multilevel modeling
				Prepregnancy BMI and childhood EI
				EI and child BMI at 15 y
Rogers et al. (2003)^20^	FR (ss)	1.5 y	XS	SEB food and nutrient differences
Rogers et al. (2002)^21^	FR (ss)	1.5 y and 3.5 y	XS and L	Fat intake quartiles association with foods and nutrients
Rogers et al. (2001)^22^	FR (ss)	1.5 y and 3.5 y	XS and L	Fat intake quartiles association
				with growth and obesity, iron status and blood lipids
Cribb et al. (2013)^23^	FR (ss)	1.5 y and 3.5 y	XS and L	Vitamin A and carotene
				Core and noncore foods
Cribb et al. (2014)^24^	FR (ss)	1.5 y and 3.5 y	XS and L	Vitamin D and calcium
				Fortification model
Sayers et al. (2012)^25^	(AA)	Mid-childhood		Vitamin D status and cortical bone
Johnson et al. (2007)^26^	FR (ss)	5 y and 7 y	XS and L	Dietary energy density associations
				with nutrients; misreporting of energy intake; tracking of intake and association with fat mass at 9 y
Johnson et al. (2009)^27^	FR (AA)	10 y	XS	Energy density and *FTO* genotype association with fat mass
Jones et al. (2010)^28^	FR (AA)	7 y; Maternal FFQ	XS	Fruit and vegetable intake
Johnson et al. (2007)^29^	FR (ss)	5 and 7 y	XS and L	Drinks description and SEB;
				association with fat mass at 9 y
Jago et al. (2010)^30^	FR (AA)	10 y; physical activity 11 y	XS	Dietary association with physical activity
Noel et al. (2010)^31^	FR (red s)	13 y; physical activity 13 y	XS	Use of measured physical activity for estimation of misreporting
More et al. (2014)^32^	FR (ss)	1.5 y and 3.5 y	XS	Portion sizes and foods consumed
Rogers et al. (2007)^33^	FR (ss)	7 y	XS	School meals dietary intake
Wright et al. (2008)^34^	(ss)	Birth to 5 y	XS	Growth standards compared
Reilly et al. (2000)^35^	(AA)	7 y	XS	Use of BMI to identify obesity
Reilly et al. (2010)^36^	(AA)	9 y	XS	BMI and waist circumference association with fat mass from DXA
Sherriff et al. (2009)^37^	(AA)	7–11 y	XS and L	Fat and lean mass association with fitness and grip strength
Reilly et al. (2010)^38^	(ss)	11 y	XS	Validation of fat mass measurements
Hughes et al. (2011)^39^	(ss)	Birth to 15 y	L	Timing of excess weight gain
Din et al. (2013)^40^	(AA)	Birth to 13 y	L	Timing of periods of weight gain
Rogers et al. (2006)^41^	(AA)	9 y	L	Birth weight and ponderal index association with fat and lean mass at 9 y
Howe et al. (2010)^42^	(AA)	Birth to 15 y	L	Δponderal index and ΔBMI association with fat mass at 15 y
Ong et al. (2009)^43^	(AA)	Girls only, birth to 9 y	L	Infancy weight gain association with BMI and fat mass, overweight
McCarthy et al. (2005)^44^	(ss)	2.5–5 y	L	Change in BMI and waist circumference
Reilly et al. (1999)^45^	(ss)	4 and 5 y	XS	Obesity prevalence
Hughes et al. (2011)^46^	(AA and ss)	3–15 y	L	Incidence of obesity
Reilly et al. (2011)^47^	(AA)	7–13 y	L	Progression from overweight to obesity
Wright et al. (2010)^48^	(AA)	7–11 y	L	Tracking of fatness and obesity
Reilly et al. (2005)^49^	(AA and ss)	7 y	XS and L	Risk factors for obesity
Sovio et al. (2011)^50^	(AA)	Birth to 10 y	MA	*FTO* genotype and BMI
Howe et al. (2012)^51^	(AA)	Birth to 10 y	L	SEB differences in height trajectories
Howe et al. (2010)^52^	(AA)	Birth to 10 y	L	SEB differences in adiposity trajectory
Howe et al. (2010)^53^	(AA)	9 y and 10 y	XS	SEB differences in CVD risk
Dorosty et al. (2000)^54^	FR (ss)	1.5 y	XS and L	Adiposity rebound and diet, association with parental obesity
Timpson et al. (2008)^55^	FR (AA)	10 y	XS	Diet association with *FTO* gene
Fraser et al. (2011)^56^	FFQ (AA)	13 y	XS	Frequency of eating fast food association with healthy foods and BMI
Noel et al. (2011)^57^	FR (red s)	10 y and 13 y	XS and L	Milk intake association with body fat percentage
Noel et al. (2013)^58^	FR (red s)	10 y and 13 y	XS and L	Flavored milk association with change in body fat percentage
Bigornia et al. (2014)^59^	FR (red s)	10 y and 13 y	XS and L	Dairy food association with body fat percentage
Cowin et al. (2001)^60^	FR (ss)	1.5 y	XS	Diet association with iron status
Cowin et al. (2000)^61^	(ss)	2.5 y and 3.5 y	XS and L	Birth weight and size, obesity association with blood lipids
Cowin et al. (2001)^62^	FR (ss)	1.5 y	XS and L	Diet association with blood lipids at 2.5 y
Rogers et al. (2006)^63^	(ss)	5 y and 7–8 y	XS and L	Height association with IGF axis
Rogers et al. (2006)^64^	FR (ss)	7 y	XS	Milk and dairy association with IGF axis
Rogers et al. (2005)^65^	FR (ss)	7 y	XS	Food and nutrient association with IGF axis
Ong et al. (2004)^66^	(ss)	7–8 y	XS and L	Insulin sensitivity and secretion
Salgin et al. (2012)^67^	(ss)	7–8 y	XS and L	Free fatty acids and insulin secretion
Shultis et al. (2005)^68^	FR (ss)	1.5 y, 3.5 y, and 7 y	L	Diet association with birth weight
Rogers et al. (2010)^69^	FFQ and FR (AA)	3 y, 7 y, and 10 y	L	Diet in relation to age at menarche in girls
Timpson et al. (2007)^70^	(AA)	10 y	XS	Genetics of bitter taste detection
Golding et al. (2009)^71^	(AA)	4 y and 10 y	L	Growth and feeding behavior in bitter “super” tasters
Emond et al. (2010)^72^	FFQ (AA)	3 y and 4 y	L	Diet and growth in autism spectrum disorder

*Abbreviations*: AA, all available subjects from the whole cohort; BMI, body mass index; CVD, cardiovascular disease; DXA, dual-energy X-ray absorptiometry; EI, energy intake; FFQ, food frequency questionnaire; FR, food record; IGF, insulin-like growth factor; L, Longitudinal; MA, meta-analysis; red s, reduced sample size excluding FR with 1 and 2 days only; SEB, socioeconomic background; ss, CIF sub-sample; XS, cross-sectional; y, age of child in years

Δ, change in

### Subjects

The ALSPAC is a birth cohort study that recruited pregnant women resident in 3 health districts surrounding the city of Bristol in the county of Avon, in southwest England, with an expected delivery date between April 1991 and December 1992 (n = 14 541 pregnancies).^73^^,^^74^ It was set up to investigate the ways in which genes and the environment, including diet, interact to affect the health, behavior, and development of children. Ethical approval for the study was obtained from the ALSPAC Law and Ethics Committee and the local research ethics committees. The cohort was population based and broadly representative, at recruitment, of the population of women with children aged <1 year in Avon.^74^ The indicators of the SEB of the family at recruitment are shown in Table 2. The children (n = 14 062 at birth; n = 13 988 alive at 1 y) have been followed using questionnaires completed by parents and the children, educational records, and hands-on assessment at dedicated research clinics.^73^ A proportion of children born in the last 6 months of the recruitment phase (equivalent to 10% of the whole cohort) was selected to take part in a substudy known as Children in Focus (CIF).^73^ Parents of these children were invited to bring their child to research clinics at intervals from age 4 months to age 5 years (n = 1432 ever attended). When the children were aged 7 years, the whole cohort was invited to attend the research clinic. At this age, an attempt was made to bolster the initial sample with eligible subjects who failed to join the study originally; 713 additional children (from 706 pregnancies) were recruited (total child cohort: n = 14 701 alive at 1 y). The clinic evaluations were repeated annually until age 15 years. The ALSPAC website contains details of all of the data that are available through a fully searchable data dictionary.^75^ Table 2 also shows the SEB of the mothers who completed a food record for their CIF child at age 3 years and the SEB of mothers of children who attended the research clinic and kept food records at ages 7 years and 13 years in comparison with the originally recruited mothers. The retained mothers have higher education attainment, are older, and have more favorable health indicators than mothers whose children did not complete the follow-ups.

**Table 2:** Socioeconomic background of the mothers recruited to the ALSPAC, including those who supplied dietary information at age 3 years for their child in the 10% subsample of ALSPAC, and those who supplied dietary information about their child at ages 7 years and 13 years

Characteristic	No. (%) of recruited mothers (n = 14 541)	No. (%) of mothers who completed food record for child at age 3 years (CIF only; n=863)	*P*-value	No. (%) of mothers who completed food record for child at age 7 years (n = 7285)	*P*-value	No. (%) of mothers who completed food record for child at age 13 years (n = 6112)	*P*-value
Level of education			<0.001		<0.001		<0.001
No school qualifications at age 16 y	3709 (25.5)	174 (20.2)		1390 (19.1)		1151 (18.8)	
School qualification obtained at age 16 y	4273 (29.4)	308 (35.7)		2384 (32.7)		1982 (32.4)	
Education beyond age 16 y	4358 (30.0)	363 (42.1)		2907 (39.9)		2499 (40.9)	
Missing	2201 (15.1)	18 (2.1)		604 (8.3)		480 (7.9)	
Maternal age at birth (years)			<0.001		<0.001		<0.001
<20	655 (4.5)	13 (1.5)		112 (1.5)		99 (1.6)	
20–24	2682 (18.4)	90 (10.4)		911 (12.5)		745 (12.2)	
25–29	5369 (36.9)	356 (41.3)		2718 (37.3)		2309 (37.8)	
30–34	3808 (26.2)	304 (35.2)		2299 (31.6)		1943 (31.8)	
≥35	1382 (9.5)	100 (11.6)		862 (11.8)		710 (11.6)	
Missing	645 (4.5)	0		383 (5.3)		306 (5.0)	
Housing tenure			<0.001		<0.001		<0.001
Mortgaged or owned	9757 (67.1)	700 (81.1)		5641 (77.4)		4742 (77.6)	
Council and housing association rented	2138 (14.7)	80 (9.3)		584 (8.0)		467 (7.6)	
Privately rented or other	1440 (9.9)	70 (8.1)		492 (6.8)		434 (7.1)	
Missing	1206 (8.3)	13 (1.5)		568 (7.8)		469 (7.7)	
Ethnicity			<0.001		<0.001		<0.001
White	11927 (82.0)	830 (96.2)		6549 (89.9)		5517 (90.3)	
Nonwhite	321 (2.2)	15 (1.7)		112 (1.5)		102 (1.6)	
Missing	2293 (15.8)	18 (2.1)		624 (8.6)		493 (8.1)	
Smoked in last trimester of pregnancy			<0.001		<0.001		<0.001
Yes	2413 (16.6)	110 (12.8)		897 (12.3)		683 (11.2)	
No	8859 (60.9)	664 (76.9)		5254 (72.1)		4468 (73.1)	
Missing	3269 (22.5)	89 (10.3)		1134 (15.6)		961 (15.7)	
Maternal prepregnancy BMI			<0.001		<0.001		<0.001
<18.5	567 (4.0)	36 (4.2)		259 (3.6)		226 (3.7)	
18.5–24.99	8564 (58.2)	584 (67.7)		4761 (65.4)		4029 (65.9)	
25–29.99	1739 (12.0)	129 (14.9)		924 (12.7)		774 (12.7)	
≥30	631 (4.3)	32 (3.7)		317 (4.4)		257 (4.2)	
Missing	3033 (20.8)	70 (8.1)		1024 (14.1)		826 (13.5)	

*Abbreviations:* BMI, body mass index; CIF, Children in Focus.

### Dietary assessment

Table 3 shows the timing and response rates for the collection of the dietary data by food frequency questionnaire (FFQ) and food record.

**Table 3:** Dietary data available for the ALSPAC mothers during pregnancy (recruited, n = 14 541^a^), the ALSPAC children (at birth, n = 14 062^b^), and a 10% subsample of children who were studied more intensively (ever attended, n = 1432) with response rates

Age of child	Sample	Type of dietary data	Response (n)	Rate (%)
32 weeks gestation	Mothers	FFQ	12 423	85.4
1.5 y	10% subsample	3-day food record	1026	71.6
3 y	Children	FFQ	10 137	69.7
3.5 y	10% subsample	3-day food record	863	60.3
4 y	Children	FFQ	9715	66.8
5 y	10% subsample	3-day food record	772	53.9
7 y	Children	FFQ	8505	58.5
7 y	Children	3-day food record	7285	50.1
9 y	Children	FFQ	8223	56.6
13 y	Children	3-day food record	6112	42.0
13 y	Children	FFQ	7079	48.7

^a^ 604 no live birth, 69 unknown outcome.

^b^ 208 multiple births.

*Abbreviation*: FFQ, food frequency questionnaire.

### Food frequency questionnaire

Maternal diet was assessed using an unquantified self-completion FFQ sent to the mothers at week 32 of gestation.^76^ It was later adapted to cover the child’s diet and was completed by parents about the child at various ages (Table 3). The parental questionnaire when the child was aged 13 years asked only about foods provided by the parents to the child; the adolescents themselves were asked to complete a separate questionnaire about foods they ate outside of the home, such as school meals, take-away foods, confectionery, and soft drinks.^77^ For the estimation of energy and nutrient intakes, the data from these questionnaires were combined. Maternal diet was assessed again 4 years after the birth of the study child using the modified FFQ. Copies of all the questionnaires used are available online.^78^ For foods/drinks not usually consumed every day, the person was asked to indicate how often the food was consumed currently, using the following options: 1) never or rarely; 2) once in 2 weeks; 3) 1–3 times a week; 4) 4–7 times a week; and 5) more than once a day. For frequently eaten foods, there were more detailed questions, such as how many cups of tea or coffee were consumed, how much milk and sugar were used in each cup, the number of slices of bread consumed each day, on average, and how many slices were spread with butter/margarine. Questions also covered the usual type of milk (full fat or other), the usual type of bread (white, brown, or wholemeal), and the usual type of spread (butter/margarine or other) consumed. To calculate the amount of each food consumed per week, the frequency questions were converted as follows: 1) 0; 2) 0.5; 3) 2; 4) 5.5; and 5) 10 times per week. Consumption of tea, coffee, bread, milk, and fat spreads was recorded on a daily rather than a weekly basis. No questions were asked about portion sizes; therefore, standard portion sizes^79^^,^^80^ tailored to the age of the person being assessed were used for the nutrient estimations. Nutrient intakes were calculated based on the frequency with which each food was consumed and the nutrient content of a portion of that food.^76^ To avoid including outliers with abnormally high or low intakes, at each age subjects with very high or very low intakes of energy were removed from the nutrient analysis after inspection of the histogram for energy intakes at that age (between 220 and 330 subjects excluded).

### Food records

Food records were collected 6 times between the ages 1.5 years and 13 years, as listed in Table 3. Diet was assessed by parental completion of a food record in the CIF subsample at ages 1.5, 3.5, and 5 years^15^^,^^16^ and in the whole ALSPAC cohort plus the new recruits at age 7 years.^17^ The parents were invited by post to record in a structured record all foods and drinks their child consumed over 3 individual days (preferably 1 weekend day and 2 weekdays, which did not need to be consecutive). They were asked to bring the completed food records to the clinics, where, when the children were aged 1.5 years and 3.5 years, but not aged 5 years and 7 years, they were interviewed briefly by a member of the nutrition team to check for completeness and to clarify any uncertainties in the records, such as cooking methods. Foods and drinks consumed were recorded in household measures. At ages 10 years and 13 years, food records were targeted for completion by the child with parental help.^18^ The child and parent were interviewed during the clinic by a nutrition fieldworker for up to 15 minutes to obtain further details about the foods consumed.^18^ If no diary had been brought to the clinic, the nutrition fieldworker carried out a 24-hour recall for the previous day. A short questionnaire accompanied the food record and provided further details to aid interpretation of the food record. For example, the volume of the usual cup used for drinks was recorded, as well as information about any dietary supplements used.

The food records including the 24-hour recalls were transformed into weights and codes linkable to the nutrient content corresponding to each of the drinks or foods consumed using DIDO software, developed by the Human Nutrition Research Unit in Cambridge, United Kingdom.^81^ Portion sizes for foods were described using household measures. Food weights were allocated based on described portion sizes; if the description was inadequate, portion size was based on weighed intake data from a national sample of similarly aged children.^80^ Food weights were also obtained from those given on packets. Composite foods and recipes that did not have an equivalent in the food tables were broken down into their component parts.

The data bank used for the nutrient analysis of the food records included the 5^th^ edition of McCance and Widdowson’s food tables^82^ and the supplements to the tables.^83–91^ Nutrient information for any foods not covered by this database was obtained from the National Diet and Nutrition Survey database^92^ or from manufacturers’ information. These data were used to generate average daily nutrient intakes and amount consumed of various groups of food. Intakes from dietary supplements were not included in the nutrient calculations. In some analyses only children with 3 complete days of food records were included as stated in the text. The energy density of the diet was investigated by dividing total food energy (kJ) by the total weight (g) of food consumed. The energy contribution from drinks was assessed separately. A measure of added or free sugars intake (equivalent to non-milk extrinsic sugars) was calculated by deducting the sugars from milk, fruits, and vegetables from total sugars.^93^ Fiber was measured as nonstarch polysaccharide; this type of fiber does not include resistant starch or lignin substances, which are part of the fiber measured by some other methods.^93^

For some analyses, foods were grouped into nutrient-rich core food groups (bread, rice, pasta; fruits; vegetables; meat, fish, eggs; milk, cheese) or nutrient-poor noncore food groups (cakes, biscuits, confectionery, savory snacks; processed meat/poultry/fish; potatoes/vegetables with fat; spreads, sauces) according to guidelines used in Australia.^94^ This was done to differentiate foods that are recommended to be eaten by children as part of a balanced diet from foods that are extra to the basic diet and tend to unbalance it. These noncore foods are sometimes referred to as discretionary foods.

### Misreporting of energy intake

For the food records, misreporting of energy intake (EI) was assessed using an individualized method that takes into account the age, sex, and body weight of the child and allows for growth and a standard level of physical activity.^95^ The ratio of reported EI to estimated energy requirement (EER) was calculated (EI:EER). Individual EERs were estimated using equations from an *Expert Consultation Report on Human Energy Requirements*.^96^ A 95% confidence interval (CI) for the accuracy of EI:EER was calculated by taking into account the amount of variation inherent in the methods used to estimate EI and EER.^97^ For example, the confidence range for EI:EER, calculated for the data at age 7 years, was 0.79–1.21, so reports of EI between 79% and 121% of EER were considered to be within the normal range of measurement error and were defined as plausible reports. Those below this cut-off were under-reports, and those above were over-reports. Slightly different cut-offs based on the age-specific data were applied at other ages.

### Energy adjustment

Energy adjustment is necessary when assessing relationships between nutrients or food groups and age-related outcomes because energy is highly correlated with most nutrients and related to body size^98^; it may, thus, obscure underlying relationships. The residuals method described by Willet has been used to adjust nutrient intakes.^98^ For food groups, the weight of food eaten has been divided by the total energy content of the diet at each age so that intakes are directly comparable between the ages and sexes. Energy adjustment provides an assessment of the quality of the diet as opposed to the quantity provided by the unadjusted data.

### Context for the ALSPAC data: reference nutrient intakes and national cross-sectional studies of diet in children

Dietary reference values for food energy and nutrients for the UK population are used to assess the adequacy of the diet in the ALSPAC children at each age.^93^ There is no published United Kingdom reference value recommended for fiber (nonstarch polysaccharide) in childhood; for adults, the recommendation is 18 g nonstarch polysaccharide per day. The Caroline Walker Trust has researched this matter^99^ and suggests that children should consume a percentage of adult intake related to their EI. They suggest 8 g nonstarch polysaccharide/4.2 MJ (1000 Kcal) should provide an adequate intake of fiber. In the United Kingdom, food-based recommendations for healthy eating are based on the Eatwell plate.^100^

The rolling program of the National Diet and Nutrition Survey (NDNS) carries out regular cross-sectional recording of diets as eaten by children aged 1.5–18 years in the United Kingdom using 4-day^101^ or 7-day^92^ weighed food records or food records described using household measures^102^ as the assessment tools. The children aged 1.5*–*4.5 years who were surveyed by the NDNS in 1992/1993^101^ have been compared with the ALSPAC children at ages 1.5 years and 3 years.^15^^,^^16^ The NDNS 1997^92^ children have been compared with the ALSPAC children at ages 7 years and 10 years.^17^^,^^18^ An update and extension of the NDNS in 2008–2012^102^ carried out over 4 years in children aged 1.5–18 years has been used to assess whether diet in children at different ages has changed since the time of the ALSPAC data collection.

### Physical activity

The children were asked to wear an MTI actigraph model 7164 (Manufacturing Technology Inc, Fort Walton Beach, FL, USA) for 7 consecutive days^103^ at ages 11, 13, and 15 years to measure their physical activity. Although these monitors cannot capture data for some activities (swimming, contact sports, cycling), they have been shown to provide a reasonable estimate of physical activity.^104^ The activity data collected were included in the analyses if there was >3 days of data with at least 600 minutes of data per day.^105^ Mean counts per minute, an indication of the volume of physical activity, was calculated. Moderate to vigorous physical activity was represented by the mean minutes per day in which there were >3600 accelerometer counts per minute.

### Anthropometric measurements

Birth weights were obtained from the medical records, and supine length was measured soon after birth by a member of the ALSPAC study team. Body weight and length at approximately ages 2, 9, and 19 months and at school entry were available from routinely collected measurements performed by health visitors as part of the child health surveillance program and were extracted from the local child health database. Anthropometric measurements were taken at each research clinic visit using standardized procedures described in each published article. In clinic visits from age 7 years, fat mass was assessed by bioelectrical impedance using a Tanita leg-to-leg body fat analyzer (Model TBF 305; Tanita, Tokyo, Japan) and at age 7 years only by an arm-to-leg impedance monitor (Bodystat 1500; Bodystat Ltd., Isle of Man, UK). A whole-body DXA (dual energy X-ray absorptiometry) scanner (Lunar Prodigy DXA scanner; GE Medical Systems, Madison, WI, USA) was used to measure body composition from age 9 years onward and provided estimates of total fat mass, lean body mass, and bone mass.

From these heights/lengths and weights, ponderal index in infancy (weight/length^3^) and BMI in childhood was calculated (weight/height^2^). Body weight, height/length, and BMI at each time point were converted to standard deviation (SD) scores (or *z*-scores) by comparison with the British 1990 growth reference^106^ using gestational age or the actual age at measurement for each individual. Weight gain was assessed by calculating the difference in *z*-scores between body weight at the beginning and end of the period, adjusting for regression toward the mean using LMS correlates from the British 1990 growth reference.^107^ Fat mass index (FMI) was calculated by dividing fat mass (in kg) by height (in m^x^) to adjust for body size.^108^ The optimal power (x) to raise height to was derived from the data so that the relation between fat mass and height was completely removed^108^; this power varied according to the age and sex of the child (e.g., 5.3 and 4.2 at age 11 y for boys and girls, respectively^109^). There is no generally accepted cut-off to define excess adiposity using either fat mass, percentage of body fat, or FMI. However, if age- and sex-specific BMI cut-offs from the International Obesity Task Force (IOTF)^110^ are used to assess the occurrence of overweight in subjects, a 20% prevalence of overweight is found. For comparability, it was assumed that an equivalent percentage of children should be defined as having excess adiposity; e.g., those children in the top quintile of log FMI were categorized in this way.

### Blood samples and blood pressure

Nonfasting venous blood samples were collected at research clinics at some time points; fasting samples were collected only in a subgroup of the children at approximately 8 years. Various biochemical markers were measured, and the methods are described in the individual papers. Systolic and diastolic blood pressures were measured at several ages using a Dinamap 9301 Vital Signs Monitor (Morton Medical, London, UK). Two right arm measurements were recorded using a cuff size appropriate for the child’s upper arm circumference, and the average was taken.

### Pubertal status and girls’ age at menarche

Pubertal status was self-reported by questionnaire at ages 11 years and 13 years using diagrams depicting the 5 Tanner Stages^111^ for pubic hair development and was found to be reliable for assessing maturation in this cohort. Data on whether or not the girls had started menstruating were collected at a clinic held just before they reached 13 years of age; the mean (SD) age of attendance at the clinic was 12.89 (0.23) years, ranging from 11.30 years to 14.34 years.^43^ The girls were asked during a measuring session in a private room if they had started menstruating and, if so, when. In total, 3751 girls attended the clinic. Data were available from 3298 girls on whether or not they had reached menarche; of these, 1637 (50%) stated that they had not.

### Maternal socioeconomic and anthropometric factors

Maternal age at delivery was calculated by subtracting the mother’s date of birth from the child’s date of birth. Information on highest maternal educational level was derived from a questionnaire sent out at 32 weeks gestation. All of the educational qualifications obtained by the mother were recorded, and her highest educational attainment was assessed on a 5-point scale: no academic qualifications; vocational training (hairdressing, catering, etc.); at least one O-level academic examination or equivalent usually taken at age 16 years; at least one A-level academic examination or equivalent usually taken at age 18 years; university degree. In some analyses, these categories have been further contracted to only 3 categories; low, no academic qualification or vocational; medium, O-level qualifications; high, A-level or degree-level qualifications. Information on maternal smoking status was collected by questionnaires sent during pregnancy and at various ages of the child^78^. Housing tenure data were collected during pregnancy; categories were as follows: owned or mortgaged; public or council rented; private rented. Mothers were asked via questionnaire during pregnancy to report their prepregnancy weight and height^78^; these values were used to calculate maternal prepregnancy BMI.

### Statistical methods

Because this review features a large number of published articles, it is not practical to list all of the statistical methods used in the individual articles. Descriptive statistics of the main variables, the likely biases, and the handling of missing data are provided individually in most of the articles cited. Where regression analyses were carried out, the factors adjusted for depended on the outcome of interest, and the variables used in each analysis are listed in the text, as appropriate. The main confounding variables for many of the analyses were the indicators of SEB listed in Table 2. In all cases, 95% CIs are quoted.

## RESULTS AND COMMENTARY

### Food and nutrient intakes throughout childhood

#### Longitudinal dietary change

The energy and energy-adjusted macronutrient intakes from the cross-sectional food records at all 6 ages previously published in separate articles are presented together in Table 4.^15–18^ For the children aged 10 years and 13 years, among whom misreporting rates were high, data from plausible reporters only were used. The full nutrient details are available in the individual articles cited in Table 4. Energy intake rose in line with the increasing size of the children, as expected; within this, the contribution from protein fell whereas that from carbohydrate rose. The percentage of energy from protein was highest at age 1.5 years, then relatively constant from age 5 years onward (decline of 1.2 [95% CI, 1.0%–1.4%] between age 1.5 years and age 3 years, n = 775; 0.7 [95% CI, 0.5%–0.9%] between age 3 years and age 5 years, n = 636; both *P *< 0.001 by paired *t*-test). The increase in energy from carbohydrate was mainly due to a rise in free sugars; the percentage of energy from free sugars rose by 3.3 (95% CI, 2.9%–3.8%) between age 1.5 years and age 3.5 years and by 1.2 (95% CI, 0.7%–1.7%) between age 3 years and age 5 years (both *P *< 0.001). Fiber intake relative to EI was very similar at each age.

**Table 4:** Mean intake of energy and fiber and the percentage of energy from macronutrients at each age of food record assessment from age 1.5 years to age 13 years

Item measured	Age at assessment		*P*-value	Age at assessment		*P*-value	Age at assessment		*P*-value
	1.5 y^a^	3.5 y^b^	(1.5 y vs 3.5 y)	5 y^c^	7.5 y^d^	(5 y vs 7.5 y)	10.5 y^e^	13 y^c^	(10.5 y vs 13 y)
							(plausible only)	(plausible only)	
No. of respondents	1026	863		772	814		4462	2227	
Plausible reporters (%)	73	69		72	76		60	35	
Energy (MJ)	4.60 [0.94]	5.65 [1.1]		6.37 [1.23]	7.06 [1.32]		8.39 [1.21]	9.90 [1.59]	
Protein (%en)	15.3 [2.3]	14.0 [2.5]	<0.001	13.4 [2.3]	13.3 [2.2]	0.998	13.3 [2.3]	13.7 [2.5]	<0.001
	(15.1–15.4)	(13.9–14.2)		(13.2–13.5)	(13.2–13.3)		(13.2–13.4)	(13.6–13.8)	
Total fat (%en)	37.4 [4.7]	36.2 [4.5]	<0.001	35.5 [4.5]	35.5 [4.4]	0.838	35.9 [4.6]	35.6 [5.2]	0.409
	(37.1–37.7)	(35.9–36.5)		(35.2–35.8)	(35.4–35.6)		(35.8–36.0)	(35.4–35.9)	
Saturated fat (%en)	18.0 [3.6]	16.1 [3.3]	<0.001	14.8 [3.0]	14.2 [2.8]	0.028	14.0 [2.9]	13.6 [3.2]	<0.001
	(17.7–18.2)	(15.9–16.3)		(14.6–15.0)	(14.2–14.3)		(13.9–14.0)	(13.5–13.7)	
Monounsaturated fat (%en)	12.0 [1.8]	11.8 [1.7]		11.9 [1.8]	11.9 [1.9]		12.2 [2.0]	11.9 [2.3]	
	(11.9–12.1)	(11.7–12.0)		(11.8–12.0)	(11.9–12.0)		(12.2–12.3)	(11.8–12.0)	
Polyunsaturated fat (%en)	4.3 [1.6]	5.0 [1.6]		5.4 [1.7]	5.5 [1.6]		5.9 [1.8]	5.8 [1.9]	
	(4.2–4.3)	(4.9–5.1)		(5.3–5.5)	(5.5–5.6)		(5.8–5.9)	(5.7–5.9)	
Carbohydrate (%en)	46.7 [6.0]	49.9 [5.0]	<0.001	51.2 [5.4]	51.1 [5.1]	0.944	50.8 [4.9]	50.6 [5.9]	0.037
	(47.1–47.7)	(49.5–50.2)		(50.8–51.5)	(51.2–51.4)		(50.7–51.0)	(50.4–50.9)	
Starch (%en)	20.8 [5.2]	23.8 [5.5]		25.4 [4.7]	26.5 [4.4]		26.6 [4.4]	26.8 [5.1]	
	(20.5–21.1)	(23.4–24.1)		(25.1–25.7)	(26.4–26.6)		(26.5–26.7)	(26.6–27.0)	
Free sugars (%en)	12.3 [6.0]	16.4 [5.9]	<0.001	17.1 [6.0]	17.4 [5.3]	0.002	18.1 [5.5]	17.9 [6.5]	0.021
	(12.0–12.6)	(15.5–16.3)		(16.7–17.5)	(17.3–17.5)		(17.9–18.3)	(17.6–18.1)	
Fiber (g of nonstarch polysaccharide)	6.6 [2.4]	8.1 [2.8]		9.3 [3.1]	10.3 [3.3]		12.1 [3.5]	14.6 [4.8]	
	(6.5–6.8)	(7.9–8.3)		(9.1–9.5)	(10.1–10.5)				

Data are presented as mean (SD) and (95% confidence intervals). These data were first published in the articles cited; for comparisons in this table, paired *t*-tests have been undertaken between certain groups.

^a^ Data from Cowin et al. (2000)^15^

^b^ Data from Emmett et al. (2002)^16^

^c^ Not published in this format previously.

^d^ Data from Glynn et al. (2005)^17^

^e^ Data from Cribb et al. (2011)^18^

*Abbreviations*: %en, percentage of energy.

Longitudinal change in EI was examined in complex statistical analyses, combining all the food record and FFQ data collected on the children between ages 3 years and 13 years.^19^ Linear-spline multilevel models were used to summarize EI trajectories through childhood and adolescence. Models estimated average and individual EI at age 3 years and linear changes in EI from age 3 years to age 7 years and age 7 years to age 13 years in 12 032 children with at least 1 food record or FFQ and were adjusted for sex, person completing food record (parent, child), source of data (FFQ, food record), and plausibility of intake. Trajectories were similar if FFQs and food records were analyzed separately or combined into one model. The FFQ estimation of EI was, on average, 74 kcal higher than that of the food records. The predicted EI at exactly 3 years was 1131 (SD, 74) kcal. The rate of increase of EI was greater between ages 3 years and 7 years (136 [SD, 16] kcal/y) than between ages 7 years and 13 years (97 [SD, 20] kcal/y).

The percentage of consumers and overall amount of selected food groups from separate publications at each age as the children progressed from preschool age to adolescence are shown in Table 5.^15–18^ Visual inspection of the data gives an insight into the relative quality of the diet over time. The data suggest that the profile of foods consumed moved away from the food-based recommendations of the UK Eatwell plate^100^ and the Australian core and noncore food concept.^94^ Within the core foods, those high in fiber (wholemeal/brown bread and whole-grain breakfast cereals) showed a relative decline in percentage of consumers, whereas white bread and other breakfast cereals showed an increase, at least up to age 7 years. Beef products were consumed by many fewer children at age 3.5 years (25%) than age 1.5 years (37%), probably a result of major publicity about bovine spongiform encephalitis in beef, which occurred just prior to collecting food records for the children aged 3.5 years; these data suggest that consumption took several years to recover back to the earlier frequency (35% by age 10 years). Beef was eaten by 45% of children aged 1.5*–*3 years in NDNS 2008–2012,^102^ confirming the recovery of intake. More children ate oily fish as they grew up, but never more than 20% of the ALSPAC children consumed it. More than 10% of children had not eaten any vegetables, and more than 15% had not eaten any fruit during the recording period at each age (Table 5). The data suggest that whole milk was gradually replaced by low-fat milk in the diets of these children. In the noncore food groups, savory snacks (crisps), biscuits, and chocolate confectionery were consumed by a majority of the children at each age, and the percentage of consumers of sugar confectionery more than doubled after age 1.5 years. Coated poultry products were consumed most often at age 7 years; they were widely used in school dinners at this time prior to reforms in school meal quality. Regular and/or diet soft drinks were consumed by a majority of the children at each age in relatively large amounts (Table 5).

**Table 5:** Percentage of children who consumed selected core and noncore food/beverage groups and the daily mean (SD) weight consumed (averaged over consumers and nonconsumers) at each age of food record assessment

Item consumed	Age at assessment					
	1.5 y^a^	3.5 y^b^	5 y^c^	7.5 y^d^	10.5 y^e^	13^c^
					(plausible only)	(plausible only)
No. of respondents	1026	863	772	814	4462	2227
Core foods and beverages^f^						
White bread (%C)	73.7	83.6	85.1	89.4	88.0	80.5
White bread (g)	20.3 (21.0)	35.6 (29.5)	48.4 (38.1)	54.3 (38.2)	64.6 (47.4)	68.4 (62.1)
Wholemeal/brown bread (%C)	34.3	32.0	26.6	22.8	24.5	21.5
Wholemeal/brown bread (g)	7.9 (15.0)	10.6 (19.6)	11.1 (23.8)	9.2 (21.6)	7.6 (21.6)	11.9 (30.7)
High fiber breakfast cereal (%C)	72.7	59.0	57.1	49.8	49.0	48.1
High fiber breakfast cereal (g)	13.9 (18.2)	13.7 (20.5)	14.1 (19.7)	15.3 (21.7)	15.6 (24.8)	21.9 (35.1)
Other breakfast cereal (%C)	45.8	65.7	63.7	57.0	51.5	38.7
Other breakfast cereal (g)	4.5 (7.5)	9.7 (10.8)	12.5 (14.9)	14.2 (17.0)	13.4 (17.8)	12.7 (21.3)
Beef (%C)	36.8	24.9	26.1	30.0	35.4	36.2
Beef (g)	9.2 (18.0)	10.3 (25.4)	8.9 (18.2)	11.7 (22.8)	21.7 (41.0)	29.0 (55.2)
Oily fish (%C)	11.4	13.6	13.5	15.4	16.5	19.7
Oily fish (g)	1.4 (5.7)	2.6 (8.5)	3.0 (9.1)	3.5 (10.1)	4.8 (14.3)	7.8 (23.9)
All vegetables (%C)^g^	91.6	83.0	83.3	88.1	85.2	86.3
All vegetables (g)^g^	39.8 (31.7)	40.5 (36.7)	44.6 (40.4)	50.7 (43.4)	65.4 (56.3)	80.1 (69.2)
All fruit (%C)	84.1	82.6	82.1	77.8	64.0	72.1
All fruit (g)	63.9 (56.5)	68.9 (63.6)	78.8 (70.8)	75.7 (74.5)	73.3 (77.8)	92.8 (106.1)
Plain potatoes (%C)	78.3	62.6	57.3	56.1	51.5	48.6
Plain potatoes (g)	29.2 (27.1)	25.4 (30.1)	25.0 (30.1)	26.8 (32.0)	34.9 (46.0)	40.8 (57.5)
Whole milk (%C)	90.2	83.7	66.6	55.2	34.5	25.1
Whole milk (g)	391 (243.9)	260.9 (231.8)	173.6 (189.8)	138.2 (189.8)	80.3 (152.0)	69.9 (174.2)
Semi/skimmed milk (%C)	14.6	33.3	44.3	54.0	61.5	65.5
Semi/skimmed milk (g)	30.4 (113.7)	76.5 (159.2)	94.0 (151.6)	122.6 (168.1)	144.7 (179.6)	178.3 (220.4)
Noncore foods and beverages^f^						
Coated poultry (%C)	10.4	24.1	29.8	34.8	27.0	16.1
Coated poultry (g)	1.6 (5.4)	5.0 (11.3)	8.3 (15.3)	10.7 (17.9)	10.4 (22.2)	8.9 (26.4)
Potatoes with fat (%C)	56.1	72.3	77.2	83.2	84.0	68.8
Potatoes with fat (g)	17.6 (23.7)	32.4 (34.1)	39.2 (34.0)	43.4 (38.1)	63.0 (54.3)	64.9 (66.4)
Biscuits (%C)	87.2	87.7	83.5	82.9	79.5	72.7
Biscuits (g)	11.0 (10.4)	15.6 (13.4)	19.1 (17.4)	19.9 (17.2)	21.6 (21.6)	24.9 (28.8)
Cakes, buns (%C)	46.6	55.4	62.3	68.6	70.0	63.8
Cakes, buns (g)	8.1 (12.4)	16.0 (21.5)	23.3 (27.6)	25.7 (27.2)	31.1 (33.4)	38.0 (44.8)
Puddings and ice cream (%C)	57.9	69.2	70.7	70.3	68.5	51.8
Puddings and ice cream (g)	24.9 (33.9)	35.8 (39.6)	39.6 (41.1)	43.8 (46.1)	48.4 (53.0)	37.9 (54.9)
Savory snacks (%C)	65.6	75.2	78.9	83.2	82.0	72.5
Savory snacks (g)	5.9 (7.2)	11.2 (10.9)	15.0 (12.1)	17.2 (12.9)	20.3 (16.3)	18.2 (18.0)
Chocolate confectionery (%C)	63.3	67.8	67.7	74.8	77.5	68.0
Chocolate confectionery (g)	6.9 (8.6)	11.6 (13.1)	11.4 (12.9)	16.3 (17.3)	20.8 (22.2)	22.8 (27.6)
Sugar confectionery (%C)	18.4	41.9	45.5	43.9	53.5	38.8
Sugar confectionery (g)	1.8 (5.3)	5.3 (9.4)	6.2 (10.7)	6.1 (10.6)	9.1 (16.1)	10.6 (25.9)
Normal soft drinks (%C)	40.3	59.0	66.7	62.3	62.4	59.5
Normal soft drinks (g)	73.4 (190.3)	116.9 (198.1)	157.2 (232.7)	147.1 (208.5)	158.5 (211.4)	215.8 (304.2)
Diet soft drinks (%C)	65.4	75.0	72.7	73.1	69.4	56.3
Diet soft drinks (g)	172.5 (248.0)	286.9 (344.7)	285.1 (345.2)	273.7 (330.2)	227.2 (263.2)	249.0 (370.1)

^a^ Data from Cowin et al. (2000)^15^

^b^ Data from Emmett et al. (2002)^16^

^c^ Not published in this format previously.

^d^ Data from Glynn et al. (2005)^17^

^e^ Data from Cribb et al. (2011)^18^

^f^ Data have not been compared by formal statistics.

^g^ Excludes baked beans.

*Abbreviation:* %C, percentage of children who consumed indicated food or drink; SD, standard deviation.

A detailed picture of the changes in the sources of energy from the food groups at the point of greatest change in the diet between age 1.5 years and age 3.5 years are given in Table 6.^23^ The decline in energy contribution from dairy foods (*P *< 0.001) probably accounted for the decreased percentage of energy from protein (Table 4), whereas the free sugars increase was likely due to the increased EI from sweet miscellaneous foods (*P *< 0.001), particularly confectionery. The contribution to EI of core foods fell from 63% to 54%, whereas the contribution of noncore foods rose from 32% to 43% (both *P *< 0.001). Of the 11% rise in energy from noncore foods, 5% was due to increased intakes of confectionery and savory snacks (crisps). These changes suggest deterioration in diet quality between the 2 ages, and Table 5 suggests that this lower-quality diet is maintained throughout childhood.

**Table 6:** Contribution to energy intake of core and noncore food groups from 3-day food records of diet consumed by the same children at age 1.5 years and age 3.5 years from the 10% subsample of the ALSPAC cohort (n=755)

Type of foods	Energy supplied by each food group (% of total)^a^		Type of foods	Energy supplied by each food group (% of total)^a^	
	At age 1.5 y	At age 3.5 y		At age 1.5 y	At age 3.5 y
*Core foods*			*Noncore foods*		
Bread, cereals, rice, and pasta			Miscellaneous		
Bread	7.1	9.2	Puddings and ice-creams	3.3	4.3
Breakfast cereals	5.6	5.6	Buns, cakes, and pastries	2.8	4.3
Pasta, rice and savories	2.7	3.4	Sweet biscuits	4.9	5.7
Group total	15.3^b^	18.2^c^	Savory biscuits	0.7	0.6
Vegetables and legumes			Confectionery	4.8	7.1
Vegetables	1.5	1.3	Crisps	2.5	4.3
Potatoes	2.1	1.7	Group total	19.1^b^	26.3^c^
Vegetable dishes	0.3	0.3	Processed meat, fish, and poultry		
Legumes	0.1	0.1	Processed meats	0.8	1.0
Group total	4.0^b^	3.3^c^	Coated chicken	0.3	0.9
Fruit			Burgers and kebabs	0.2	0.2
Fruit	4.4	3.4	Sausages	1.1	1.4
Fruit juice	1.5	1.8	Meat pies	0.8	0.9
Group total	5.9^b^	5.2^c^	Coated and fried fish	1.0	1.3
Yogurt, cheese, and milk			Group total	4.3^b^	5.8^c^
Milk	25.1	15.6	Vegetables		
Yogurt	4.0	3.2	Fried/roast potatoes	2.9	4.3
Cheese	2.3	2.6	Baked beans	1.2	0.9
Group total	31.4^b^	21.4^c^	Group total	4.1^b^	5.3^c^
			Spreads, soup, and sauces	
Meat, fish, poultry, and eggs			Fat spreads	3.3	4.3
Meat	2.7	2.4	Soup	0.3	0.3
Fish	0.5	0.5	Milk-based sauces	0.2	0.1
Poultry	1.2	1.6	Tomato-based sauces	0.1	0.2
Eggs and egg dishes	1.2	1.1	Other sauces	0.5	0.6
Group total	5.7	5.6	Group total	4.3^b^	5.5^c^
Core foods total	62.8^b^	53.8^c^	Noncore foods total	31.8^b^	42.8^c^

Modified from Cribb VL et al. (2013)^23^ with permission.

^a^ The percentage of energy from core and noncore foods does not add up to 100% because a few energy-containing foods have not been included in the classification.

^b^^,^^c^ Groups with different letters show a significantly different energy contribution between ages by paired *t*-test (*P *< 0.001).

#### Comparisons with dietary recommendations and cross-sectional dietary intake data from representative samples of UK children

The profile of fat intake improved slightly over childhood (Table 4), with a fall in the overall contribution of fat to energy toward the recommended level of <35%.^15–18^ This was mainly due to a fall in energy from saturated fat, although intake was still above the recommendation of 10% of energy. The amount of free sugars consumed at all ages was much higher (Table 4) than the maximum 10% of energy recommended.^93^ Fiber (nonstarch polysaccharide) intakes were approximately 75% of the recommended adequate intake at each age throughout childhood (Table 4); e.g., an adequate intake for children aged 3 years is approximately 10.8 g nonstarch polysaccharide/day, but the mean intake in these children at age 3.5 years was 8.2 g/day.^16^^,^^99^

Most micronutrient intakes were adequate at all ages when compared with the recommendations,^93^ the exceptions being low intakes of vitamin D and iron. Average intakes for iron were below the recommended amounts at all ages in childhood.^15–18^ Current recommendations for Vitamin D cover only children aged 3 years or below.

Intakes of both foods and nutrients were similar to those in comparable age groups in NDNS 1992/1993,^101^ NDNS 1997,^92^ and NDNS 2008–2012.^102^ Full comparisons are presented in each of the ALSPAC publications covering childhood diet.^15–18^ In the most recent survey, the children aged 1.5*–*3 years in NDNS 2008–2012 consumed more fruit than the ALSPAC children; 93% ate fruit in 2008–2012 compared with approximately 83% in ALSPAC in 1993–1996. This was due to fruit being provided to children in school breaks free of charge. In NDNS 2008–2012, free sugars contributed 11.9% of the energy in the youngest group, increasing to 14.8% and 15.8% at ages 4*–*10 years and 11*–*18 years, respectively,^102^ paralleling the ALSPAC findings. Furthermore, 31% of children aged 1.5*–*3 years in the NDNS ate sugar confectionery, increasing to 49% of children aged 4*–*10 years,^102^ which is very similar to the ALSPAC levels (see Table 5). This suggests that high sugar consumption is still prevalent in children.

#### Dietary differences in relation to family socioeconomic background

The quality of the diet in relation to family SEB was investigated in preschool children at ages 1.5 years and 3 years.^20^ The strongest associations for diet were with maternal education: the nutrient differences found were lower intakes of nonstarch polysaccharide and many micronutrients and higher intakes of free sugars in children of the mothers with low compared with high education. Differences in foods consumed were split mostly along core and noncore food lines: cheese, yogurt, fruit, and fruit juice were consumed less often and chocolate, savory snacks, meat products, and fried potatoes were consumed more often by children of mothers with low compared with high education (all *P *< 0.001).

At age 10 years, child’s dietary differences relating to maternal education (low [n = 832], medium [n = 1472], and high [n = 1820]) were examined again.^18^ There was no difference in overall EIs between the education groups; however, energy-adjusted fat intake was slightly higher (by 3.1%) and carbohydrate and protein intake slightly lower (both by ∼2%) and fiber intake lower (by 6.1%) in low compared with high maternal education groups (all *P *< 0.001). Intake of free sugars was high in all groups (∼85 g/d, which is twice the recommended maximum). The differences in some micronutrients were substantial, e.g., vitamin C was 23% lower, carotene was 15% lower, retinol was 12% lower, and sodium was 3% higher in the low compared with the high maternal education groups. Food group differences were also evident, with the core foods more likely to be consumed by children of mothers with high educational attainment; in the high maternal education group vs the low maternal education group, wholemeal bread was consumed by 30.1% vs 18.5%, oily fish was consumed by 22.3% vs 10.6%, cooked vegetables were consumed by 82.7% vs 73.6%, and fresh fruit was consumed by 81.7% vs 59.9%, respectively (all *P *< 0.001). Conversely, noncore foods were consumed by a higher proportion of the low maternal education group compared with the high maternal education group; meat pies and pasties were consumed by 20.9% vs 14.7%, fried potatoes and chips were consumed by 84.9% vs 73.2%, crisps/savory snacks were consumed by 84.5% vs 78.0%, and diet drinks were consumed by 76.0% vs 61.0%, respectively (all *P *< 0.001).^18^ The overall trend in both nutrients and foods was toward a more energy-dense, nutrient-poor diet as the educational attainment of the child’s mother decreased.

#### Individual nutrients

##### Dietary fat

Recommendations about the amount of dietary fat that is suitable for preschool children are confusing. An investigation was made into differences in nutrient, food group intakes, and growth between children divided into quartile groups according to their intake of energy from fat.^21^ The average contribution to energy from fat across the quartiles rose from 31.2% (SD, 2.8) in the lowest quartile to 43.1% (SD, 2.2) in the highest quartile at age 1.5 years and from 30.4% (SD, 2.5) to 41.8% (SD, 2.0), respectively, at age 3.5 years. There was an increase in EI between the lowest and the highest fat intake quartiles (by 7.4% at age 1.5 years and 5.5% at age 3.5 years; both *P *< 0.001). However, there was a drop in total carbohydrate intake between the lowest and the highest fat intake quartiles, which was driven by a drop in intake of free sugars (by 44% at age 1.5 years and 29% at age 3.5 years; both *P *< 0.001). Some vitamins and minerals increased with increasing fat intake quartile, particularly retinol equivalents and zinc; however, intakes of iron and most water-soluble vitamins fell. Vitamin C intake was 49% and 39% lower in the highest than the lowest quartile of fat intake at ages 1.5 years and 3.5 years, respectively, whereas the equivalent differences for iron intake were 23% and 19% lower (all *P *< 0.001). The foods increasing with rising fat intake were whole milk, meat products, cheese, and crisps/savory snacks (all *P *< 0.001). Those decreasing with increasing fat intake were semi-skimmed milk, fruit, fruit juice, breakfast cereal, and fish (all *P *< 0.001). There were no differences in growth among children in the 4 dietary fat quartiles; height and BMI at age 1.5 years and age 2.5 years were not different between quartiles at age 1.5 years, and height and BMI at age 3.5 years and age 5.0 years were not different between quartiles at age 3.5 years.^22^ There was also no difference in the proportion of children in each quartile that were at either extreme of height or BMI at any of the ages investigated. These results suggest that within the range of fat intakes normally consumed by United Kingdom preschool children, there is no evidence of an effect on growth or obesity development of increasing the proportion of EI coming from fat in the diet.

##### Vitamin A

The sources of vitamin A (retinol equivalents) in the diet and changes in intake between age 1.5 years and age 3.5 years were examined longitudinally (n = 755 with food records at both ages).^23^ The diet was categorized by core and noncore foods, and there was evidence that the quality of the diet deteriorated with age.^23^ Between age 1.5 years and age 3.5 years, the dietary intake of vitamin A decreased by 56 µg/day (95% CI, −17 to −95 µg/d; *P *= 0.005) mainly due to decreased intake of whole milk (390 g/d at age 1.5 y; 261 g/d at age 3.5 y) and increased intake of energy-dense, nutrient-poor foods such as puddings, cakes, biscuits, confectionery, and crisps. The contribution to energy of these foods rose from 19.1% to 26.3% (*P *< 0.001), with very little contribution to vitamin A intake. These analyses highlight the importance of feeding young children foods that are rich in nutrients in place of energy-dense, nutrient-poor foods.

##### Vitamin D

A parallel analysis investigated vitamin D and calcium intakes and changes with age between 1.5 and 3.5 years.^24^ Overall, vitamin D intakes were low; all children had intakes below the UK dietary recommendations at both ages. Calcium intakes decreased between the 2 ages due to reduced milk/dairy consumption. Children in the lowest quartile for vitamin D intake at 1.5 years were twice as likely to remain in that quartile at 3.5 years (odds ratio [OR], 2.35; 95% CI, 1.56–3.55). Only 18% of children consumed supplements containing vitamin D at age 1.5 years, falling to 11% at age 3.5 years. In NDNS 2008–2012, young children obtained only a quarter of the vitamin D intake recommended to be obtained from diet and only 12% took dietary supplements^102^; thus, there is a continuing problem in the United Kingdom. Whether fortification of foods could be the answer to this problem was examined; the theoretical intakes from different fortification regimens tested suggested that milk fortified at 2 µg vitamin D/100 g would provide most preschool children with adequate but not excessive intakes.^24^ Later work in the ALSPAC looking at plasma concentrations in mid-childhood of vitamin D (25-hydroxyvitamin-D_2_ and 25-hydroxyvitamin-D_3_) in relation to cortical bone measures found positive associations with D_3_ only, suggesting that in supplementation or fortification, D_3_ should be used in preference to D_2_.^25^ These 2 papers give valuable insights into how the lack of vitamin D in the diets of the majority of UK residents could be tackled.

##### Iron

Iron is a nutrient that is essential to a child’s growth and development. Several of the ALSPAC articles reporting on diet in childhood identify that a sizeable proportion of children, particularly girls,^15–18^^,^^22^ have iron intakes that are below recommended amounts throughout childhood. This is especially worrying for girls because their iron needs increase as they enter adolescence due to increased losses from menstruation.

#### Dietary energy density

The food energy density of the diet was investigated in the CIF subsample at age 5 years and age 7 years with the energy contribution from drinks considered as a separate variable in the analysis.^26^ Overall, the energy density of the diet was approximately 8.5 at age 5 years and approximately 8.8 kJ/g at age 7 years, and there was relatively strong tracking between the 2 ages (intraclass correlation coefficient: 0.62; 95% CI, 0.55–0.68), suggesting that children with a highly energy-dense diet at age 5 years were likely to have a diet with a high energy density at age 7 years. There was no evidence of an association between dietary energy density and EI at age 5 years, but at age 7 years there was a weak association (*r *= 0.15), suggesting that diets with a high energy density at 7 years have a slightly increased energy content. At both ages, energy-dense diets have higher fat and lower fiber content than diets that have a low energy density.^26^

The energy density of the diets at age 10 years was calculated (without drinks) for another study.^27^ Mean energy density was 8.76 (SD, 1.63) kJ/g, and at this age, increasing energy density was strongly associated with increasing overall EI (*r *= 0.22; *P *< 0.0001). Energy density was lower among under-reporters than plausible reporters (8.45 [SD, 1.67] kJ/g vs 8.87 [SD, 1.56] kJ/g; *P *< 0.0001), underlining the importance of accounting for reporting status in any analyses using these data. The data suggest that the positive relationship between energy density and EI becomes stronger as children grow up.

#### Individual food groups

##### Fruit and vegetable intakes

Determinants of fruit and vegetable intakes were investigated using the whole ALSPAC cohort of children aged 7 years who provided food records (n = 7285).^28^ The possible determinants assessed were mother’s consumption of fruit and vegetables (from an FFQ completed when the child was aged 4 y), provision of fruit and vegetables at meals in the home, child’s eating behaviors (liking to try a variety of foods, choosiness, and enjoyment of food), maternal education, and family expenditure on food per head.^28^ Total fruit consumption in boys and girls was 123 and 133 g/day, and total vegetable consumption was 71 and 72 g/day, respectively, confirming that boys consumed less of fruit than girls. However, there was no difference between boys and girls in the determinants of intake, so they were combined for this analysis. For children to achieve the recommended 5 portions of fruit and vegetables per day, total intake should be around 45 g/MJ/day (equating to 315 g/d for a 7-year-old child). The average intake in these children was just above half of this amount.

For fruit intake (on average, 18 g/MJ/d), in a model that only included items that were independently related to fruit intake (n = 5259), 14% of the variance in intake was explained.^28^ Providing fruit as part of everyday meals compared with not doing so had the strongest independent association (adjusted difference, 7.2; 95% CI, 6.9–8.3 g/MJ/d higher intake; *P *< 0.001); there were also associations for the highest tertile of maternal fruit consumption compared with the lowest (adjusted difference 6.3; 95% CI, 5.3–7.3 g/MJ/d; *P *< 0.001), high maternal educational attainment compared with low (adjusted difference 5.7; 95% CI, 4.6–6.8 g/MJ/d; *P *< 0.001), the highest level of expenditure on food compared with the lowest (adjusted difference 3.8; 95% CI, 2.4–5.0 g/MJ/d; *P *< 0.001), and the child not being choosy about food compared with being very choosy (adjusted difference 2.8; 95% CI, 1.6–3.9 g/MJ/d; *P *< 0.001). The child liking variety or enjoying food was not associated with fruit intake.

For vegetable intake (on average, 10 g/MJ/d), in a similar model (n = 5208), child factors were much more important than they had been for fruit intake, all variables explained 9% of the variance.^28^ The child not being choosy compared with the child being very choosy was associated with the highest difference (adjusted difference, 2.6: 95% CI, 1.9–3.3 g/MJ/d higher intake; *P *< 0.001); the child liking compared with not liking to eat a variety of foods also had a strong association (adjusted difference, 1.5; 95% CI, 0.9–1.9 g/MJ/d; *P *< 0.001). Maternal factors included regularly providing vegetables at family meals compared with not doing this (adjusted difference, 1.7; 95% CI, 1.3–2.2 g/MJ/d; *P *< 0.001), mother being in the highest tertile of vegetable intake compared with the lowest (adjusted difference, 1.7; 95% CI, 1.2–2.1 g/MJ/d; *P *< 0.001), and mother having a high compared with a low educational attainment (adjusted difference, 1.2; 95% CI, 0.7–1.7 g/MJ/d; *P *< 0.001). Expenditure on food and child enjoyment of food were only weakly associated (*P *< 0.05).^28^ In summary, maternal example and regular provision are important in determining children’s intakes of fruits and vegetables, and a child’s eating behavior adds another dimension, particularly in relation to vegetable eating.

##### Drinks in the diet

The type and volume of drinks consumed was investigated in detail in the CIF subsample at age 5 years and age 7 years.^29^ The analysis focused on sugar-sweetened beverages but assessed other drinks as well. At both ages, the most popular drink was milk (median intake, 257 and 242 g/d at age 5 y and age 7 y, respectively); the next most popular drinks were artificially sweetened diet soft drinks (127 and 140 g/d), with water (56 and 75 g/d) and sugar-sweetened beverages (57 and 67 g/d) at similar amounts. There was a bias in consumption of some of the drinks by maternal education; at both ages, children with degree-educated mothers consumed the largest amounts of fruit juice and water and the smallest amounts of diet soft drinks; children whose mothers had vocational training consumed the largest amount of diet drinks (all *P *< 0.01).^29^ Drinking diet soft drinks may disrupt appetite control mechanisms because sweetness is disassociated from energy content.^112^ Child intakes of sugar-sweetened beverages and milk did not differ according to maternal education groups.

#### Physical activity and diet

Objectively measured physical activity was available at age 11 years; this was combined with dietary intake at age 10 years to test whether there was a demonstrable relationship between diet and physical activity (n = 5134 with diet and physical activity; n = 3684 if restricting to plausible reporters only).^30^ Physical activity was higher in boys than in girls (in the full sample; means of 664 and 552 counts per minute, respectively). The amount of time spent in moderate to vigorous physical activity was also higher in boys than in girls (mean of 28 min compared with 18 min/d on a weekday; all *P *> 0.005). Boys recorded higher energy consumption than girls (mean of 1952 compared with 1769 kcal/d); however, boys consumed a lower weight of fruit and vegetables than girls (mean of 136 compared with 147 g/d).^30^ Only weak associations between physical activity and dietary intake were found, which differed between boys and girls: the most consistent associations were in boys, with total energy and percentage of energy from carbohydrate positively related (both *P *= 0.007) and percentage of energy from fat negatively related (*P *= 0.021); the only association in girls was with fruit and vegetable consumption, which was positively related (*P *= 0.027). Using plausible reporters only did not change the associations greatly. It is possible that associations between diet and physical activity would have been stronger if diet had been measured at the same time as physical activity; however, these data suggest that the 2 behaviors are relatively independent of each other.

#### Plausibility of dietary energy reporting

The plausibility of the dietary EIs recorded at each age was investigated in relation to predicted energy requirements, and in general, the frequency of likely under-reporting of EI increased and the frequency of likely over-reporting of EI decreased with age.^15–18^ The frequency of misreporting was compared in the same children at age 5 years and age 7 years (plausible reporters: 72% and 76%, respectively).^26^ There was evidence of tracking between these ages (intraclass correlation coefficient: 0.41: 95% CI, 0.34–0.48), suggesting that those who under-reported EI at age 5 years were slightly more likely to under-report at age 7 years as well.^26^ In the food records collected at age 10 years^18^ and age 13 years, the frequency of under-reporting was high (36% and 62%, respectively) but in line with NDNS 1997^92^ frequency for similarly aged children. At age 10 years, children who were overweight or obese were more likely than those of normal weight to under-report their intake (*P *< 0.001).^18^ There were differences in numbers of consumers and average intakes for particular foods, with under-reporters recording lower intakes than plausible reporters of some core foods (rice and pasta, whole milk; in girls only: cooked vegetables; in boys only: fresh fruit [all *P *< 0.001]) and some noncore foods (biscuits, cakes, puddings, chocolates, sweets, sweet spreads, and sugar-sweetened beverages in both sexes [all *P *< 0.001]). These data suggest that particular types of noncore, energy-dense foods tend to be under-reported.

A further complication in the identification of misreporting of dietary intakes is the possibility that differences in physical activity between individuals are distorting associations between diet and biological outcomes.^31^ At age 13 years, assessment of both diet and physical activity with reasonable objectivity was available; therefore, an investigation was made of whether the inclusion of measured physical activity in equations used to assess diet plausibility would provide clearer relationships.^31^ Three methods for predicting energy requirements were used: one allowed for standard low physical activity, the second calculated an individual’s physical activity from prediction equations, and the third used measured minutes of MPVA; all methods included an allowance for the age, sex, and size of the child. The frequency of plausible reporting was very similar among the methods (∼40%), but the use of measured physical activity gave much lower estimates of under-reporting (37.1%) and higher estimates of over-reporting (20.4%) compared with the first 2 methods (under-reporting 51.5% and 51.8%; over-reporting 7.7% and 10.3%, respectively). For all 3 methods, under-reporters had higher mean BMI and waist circumference than plausible or over-reporters, as well as a higher percentage body fat (all *P *< 0.001). Conversely, percentage lean body mass was lowest in under-reporters and highest in over-reporters (all *P *< 0.001).^31^ Because these 13-year-old children were mostly responsible for reporting their own diet with minimal parental help, it is very likely that they missed some foods; therefore, high frequency of under-reporting would be expected. The fact that using the measured physical activity to assess misreporting resulted in a shift toward over-reporting suggests that total physical activity was underestimated with this method. Further work should find ways of incorporating assessment of light physical activity, as well as moderate to vigorous physical activity, into this method to improve estimates of total physical activity.

#### Achieving healthy diet recommendations

The dietary data collected at 1.5 and 3.5 years was used to inform a study that aimed to provide appropriate portion size ranges and a practical food plan that covered all food and nutrient recommendations for use with children aged 1–4-years.^32^ Food and portion size information from the NDNS was used^80^ in combination with the ALSPAC data. To design a food plan to provide an adequate nutrient content within the recommended energy requirements for children, it was necessary to use foods with a medium or high nutrient density. The inclusion of energy-dense foods with poor nutrient content led to unbalanced nutrient-to-energy content in the food plan, so these foods had to be kept to a minimum. It proved impossible to incorporate enough vitamin D–containing foods to cover the recommendations, implying that vitamin D–deficient diets are likely to be the norm.^32^

In the mid-2000s, there was a great deal of controversy about the nutritional content of school meals. Children with food records for either packed lunches (n = 410) or school dinners (n = 211) from the CIF sub-study of 7-year-olds were identified,^33^ and the quality of school meals as consumed was examined. In general, packed lunches had a less good nutrient profile than school dinners, with their saturated fat and sugar content being higher and their micronutrient content being lower. Both types of meal were inadequate when compared with nutrient guidelines.^93^ School dinners in the United Kingdom have been improved since these meals were recorded in 2000. The packed lunches recorded were compared with food-based guidelines of what a school meal should contain^99^: 1 item from each core food group (starchy foods; fruits; vegetables; milk or dairy; meat, fish, or protein alternative). Only 3.5% contained all 5 groups; 44.3% contained <2 of the groups; 31% did not include a protein food.^33^ There was a fruit in only 41% and a vegetable/salad item in only 16% of the packed lunches. Taken as part of the whole days’ intake, the packed lunch eaters had a slightly higher overall intake of energy-adjusted saturated fat and sugar, even after controlling for maternal education (both *P *= 0.014); they also had a lower potassium intake but higher selenium intake (both *P *< 0.001) than school dinner eaters. This work highlights the fact that provision of nutritionally adequate cooked school dinners is needed and suggests that this should be accompanied by an education campaign for parents regarding what constitutes a nutritious packed lunch.^33^

#### Growth and obesity development

##### Sensitivity of assessment methods

When assessing growth and obesity development in childhood, it is necessary to standardize weight, height, and BMI against growth reference curves. Most analyses using ALSPAC growth data have used the UK 1990 growth references;^106^ however, new growth standards for children up to age 5 years were published by the World Health Organization in 2006,^113^ and the effect of using these in place of the UK reference for growth monitoring was investigated.^34^ Using the World Health Organization 2006 standards resulted in fewer ALSPAC infants being classified as underweight in the first year and more ALSPAC preschool children being classified as overweight (at age 2 y OR for overweight, 1.74; 95% CI, 1.20–2.51 and at age 5 y OR, 1.35; 95% CI, 1.02–1.78). It is essential to specify which growth reference has been used in any study investigating growth and obesity development in children.

Two methods of identifying obese children using BMI have been used in the ALSPAC, and at age 7 years the opportunity was taken to investigate the specificity (tendency to identify a nonobese child as obese) and sensitivity (failure to identify an obese child as obese) of these 2 methods against a measure of fatness derived from arm-to-leg bioimpedance.^35^ BMI obesity cut-off at the 95^th^ percentile based on the UK 1990 growth reference^106^ gave a false-positive rate of 6% (specificity) and a false-negative rate of 12% (sensitivity). The optimum cut-off with the highest specificity and sensitivity (8% each false-negative and false-positive rates) was the 92^nd^ percentile. There was no difference between boys and girls in sensitivity and specificity of these cut-offs. However, when the IOTF cut-offs for obesity (equivalent to BMI of 30kg/m^2^ in adults)^110^ were used, there was low sensitivity and a difference in sensitivity between boys and girls, with a false-negative rate of 54% in boys and 28% in girls (*P *< 0.01). Specificity was very high and not different between the sexes. Therefore, use of the IOTF cut-off would likely underestimate obesity prevalence, and this underestimation would be greater in boys than girls. When IOTF cut-offs for overweight (equivalent to BMI of 25–30 kg/m^2^ in adults) were used, sensitivity was much higher (3%–10% false-negative rate), but specificity was reduced, particularly in girls.^35^ It is very important to be clear about the reference data used when comparing data from different studies, and these data suggest that in this cohort, at least, the use of the UK 1990 growth reference may be more informative.

A separate analysis investigated whether measuring waist circumference might provide a better method of assessing fatness than measuring BMI.^36^ Data collected using dual x-ray absorptiometry (DXA) at age 9 years was used to assess fat mass, and high fat mass was determined as being in the top decile of fat mass in each sex separately. Receiver operator characteristics were assessed for BMI and waist circumference *z*-scores. The area under the curve and specificity were slightly higher for BMI than waist circumference in both sexes (all *P *> 0.05).^36^ Thus, waist measurements have no advantage over BMI for the detection of fatness, at least at age 9 years.

From bioimpedance measurements (leg-to-leg) collected at 4 ages between age 7 years and age 11 years an index for lean and fat mass was calculated using residuals from linear regression models in >7000 children at each age.^37^ These indices incorporated the height and age of the child at measurement and were calculated separately for each sex. To test whether these indices had any functional meaning, their relationship with cardio-respiratory fitness and grip strength was assessed. In both sexes at age 9 years, lean mass index but not FMI (*z*-score) was associated with cardio-respiratory fitness (partial correlation coefficient [*r*] for lean mass index: in boys, 0.20 [95% CI, 0.15–0.25]; in girls, 0.26: [95% CI, 0.22–0.30]). BMI *z*-score showed a slightly weaker relationship with cardio-respiratory fitness (in boys, 0.11 [95% CI, 0.06–0.16]; in girls, 0.17 [95% CI, 0.13–0.21]). Very similar relationships were found with grip strength at age 11 years. These results suggest that bioimpedance-derived lean mass index has functional significance and that FMI, thus derived, is more likely to be a measure of nutritional status than of function.

The reliability of DXA and bioimpedance measures of fat mass were tested in a study of 176 children aged 11 years in the ALSPAC whose body fatness was measured using DXA and bioimpedance. These results were compared with those from labeled deuterium dilution, a gold-standard method.^38^ Fat mass from deuterium dilution measurements differed from fat mass calculated for the other methods: in boys, fat mass from deuterium dilution was 9.8 (SD, 6.1) kg; for DXA, the bias was +0.9 (limits of agreement, −2.2 to + 4.1 kg); for bioimpedance, the bias was −5.2 kg (limits of agreement, −10.8 to +0.5 kg): in girls, fat mass from deuterium dilution was 12.1 (SD, 7.7) kg; for DXA, the bias was +1.2 (limits of agreement, −1.9 to + 5.1 kg); for bioimpedance, the bias was −0.2 kg (limits of agreement, −5.5 to +5.1 kg). There were also differences in slope and intercept between the methods. The limits of agreement and regression analysis suggested that errors in assessment of fat mass can be very large by either DXA or bioimpedance when compared with deuterium dilution and can differ between the sexes.^38^ However, the study concludes that both of these methods provide adequate estimates of relative fatness for use in large studies such as the ALSPAC.

##### Childhood growth

The timing of growth measured by weight gain was investigated to ascertain when excess weight gain was most likely to occur.^39^ Standardized measurements were available at various ages between birth and 15 years in 625 children. At birth, mean weight *z*-score was 0.12 (SD, 0.97) in comparison with the UK 1990 growth reference data. The mean weight *z*-score had increased to 0.22 (SD, 1.03) at age 12 months and remained near to this level at each age through 7 years. By age 9 years, it had increased to 0.37 (SD, 1.03) and by age 11 years to 0.54 (SD, 1.03), staying around this level at each age through 15 years. The mean BMI *z*-score was very similar to the weight *z*-score from age 12 months (0.20 [SD, 0.99]) through age 5 years but showed a reduction at age 7 years to 0.13 (SD, 1.06), followed by an increase at age 9 years to 0.34 (SD, 1.10), which remained through age 15 years.^39^ The weight gain data suggest there is a second period of fast weight gain beyond infancy that occurs after age 7 years and before age 11 years. The BMI data demonstrate the occurrence of an adiposity nadir prior to age 7 years and a rebound in the majority of children after age 7 years and before age 9 years.

A separate analysis with the main aim of comparing weight gain in the 5% of infants who grew slowly in the first few months of life with the rest of the cohort (95%) used all available data at each age and calculated conditional weight and length/height gain.^40^ This confirmed a period of fast growth in infancy in the normally growing part of the cohort (conditional gain z-score from age 2 months to age 9 months for weight, 0.17 [95% CI, 0.14–0.19], n = 10992; for length, 0.20 [95% CI, 0.17–0.24], n = 8638), then maintenance of weight status up to age 7 years and fast growth between age 7 years and age 10 years (for weight, 0.53 [95% CI, 0.49–0.56], n = 5301; for height, 0.44 [95% CI, 0.41–0.48] n = 5283), slowing slightly between age 10 years and age 13 years (for weight, 0.34 [95% CI, 0.30–0.39], n = 4539; for height, 0.38 [95% CI, 0.34–0.42], n = 4527). These 2 analyses confirm the later growth spurt after age 7 years and before age 11 years, and the BMI trajectory analysis^42^^,^^51^^,^ corroborates that, on average, BMI starts to increase (the adiposity rebound) at approximately age 7 years. This may be a time when interventions aimed at reducing excessive weight gain in children would be effective.

Birth weight and ponderal index at birth were positively associated with lean body mass at age 9 years in both sexes in 7336 ALSPAC children with DXA measurements of body composition.^41^ Both were also positively associated with fat mass, such that the association with birth weight was equivalent to a 2%–3% increase in fat mass per 1 SD increase in birth weight and the association with ponderal index was an approximately 7% increase in fat mass per 1 SD increase in ponderal index. These associations were adjusted for gestational age, current height, and SEB. The results suggest that the well-known relationship between birth weight and BMI reflects increases in both lean and fat tissue and that ponderal index at birth is a better predictor of adiposity than birth weight. A further investigation used ponderal index in the first 2 years and BMI from age 2 years to age 10 years in a longitudinal analysis using random-effects linear spline models and having DXA-assessed fat mass at 15 years as the outcome (n = 4601).^42^ Increases over time of both ponderal index and BMI were associated with greater fat mass at age 15 years (Table 7). The period of BMI change most strongly associated with fat mass at age 15 years was ages 2–5 years for girls and ages 5–5.5 years for boys (Table 7). Associations with cardiovascular risk factors were strongest for BMI change between age 8.5 years and age 10 years and were largely mediated by fat mass at age 15 years and not evident for ponderal index changes from age 0 to age 2 years.

**Table 7:** Ponderal index/body mass index trajectories from birth to age 10 years and their association with Ln of DXA-assessed total body fat mass at age 15 years, with multiple imputations, adjusted for age, previous periods of ponderal index/body mass index change, and confounders

Ponderal index/BMI change period	Logged DXA-assessed fat mass (95% CI) ^a^
Boys (n = 2181)	
Ponderal index at birth	0.038 (−0.004–0.079)
Ponderal index change 0–2 m	0.109 (0.067–0.152)
**Ponderal index change 2–24 m**	**0.164 (0.048–0.281)**
**BMI change 2–5 y**	**0.172 (0.134–0.209)**
**BMI change 5–5.5 y**	**0.558 (0.511–0.605)**
**BMI change 5.5–6.5 y**	**−0.259 (−0.298 to −0.220)**
**BMI change 6.5–7 y**	**−0.491 (−0.612 to −0.370)**
BMI change 7–8.5 y	0.446 (0.343–0.549)
BMI change 8.5–10 y	0.232 (0.165–0.299)
Girls (n = 2420)	
Ponderal index at birth	0.093 (0.054–0.131)
**Ponderal index change 0–1 m**	**0.102 (0.058–0.146)**
Ponderal index change 1–4 m	**0.218 (0.171–0.264)**
**Ponderal index change 4–24 m**	**0.200 (0.126–0.273)**
**BMI change 2–5 y**	**0.306 (0.271–0.342)**
**BMI change 5–5.5 y**	**0.301 (0.264–0.337)**
**BMI change 5.5–6.5 y**	−**0.244 (−0.286 to** −**0.203)**
BMI change 6.5–7 y	−0.101 (−0.178 to −0.024)
BMI change 7–8.5 y	0.222 (0.180–0.263)
BMI change 8.5–10 y	−0.063 (−0.139 to −0.012)

Reproduced from Howe LD, et al. (2010)^42^ with permission.

^a^ All variables were standardized prior to analysis. Coefficients represent the SD change in DXA-assessed total body fat mass associated with a 1 SD increase in the rate of ponderal index/BMI change. Bold text indicates that ponderal index/BMI is, on average, declining during that period. Adjusted for age, previous periods of ponderal index/BMI change, height, height squared, sex, maternal and partner education, household social class, maternal age, height, gestational age at birth, maternal and partner BMI, maternal and partner smoking during pregnancy, age at clinic attendance, and pubertal stage at measurement of outcome. Ponderal index/BMI change periods: BMI change 2–5 years: 24 and 60 months for boys, 24 and 56 months for girls. BMI change 5–5.5 years: 60 and 65 months for boys, 56 and 67 months for girls. BMI change 5.5–6.5 years: 65 and 75 months for boys, 67 and 73 months for girls. BMI change 6.5–7 years: 75 and 81 months for boys, 73 and 79 months for girls. BMI change 7–8.5 years: 81 and 103 months for boys, 79 and 105 months for girls. BMI change 8.5–10 years: 103 and 120 months for boys, 105 and 120 months for girls.

*Abbreviations:* BMI, body mass index; CI, confidence interval; DXA, dual-energy X-ray absorptiometry.

In girls only, at age 9 years an analysis investigated associations between infancy weight gain and later growth.^43^ In linear regression models, current weight, height, and BMI were positively associated with weight gain in infancy between age 0 and age 2 months, age 2 months and age 9 months, and age 9 months and age 19 months (all *P *< 0.01). However, FMI was only associated with weight gain in the first 2 periods (regression coefficients adjusted for height at 9 y and maternal education, 0–2 mo: β = 0.15 ± 0.06 [*P* = 0.01]; 2–9 mo, β = 0.09 ± 0.04 [*P* = 0.001]). In logistic regression analysis, each 1 U increase in weight SDS between age 0 and age 9 months was associated with a 48% increased risk of overweight at age 9 years (OR, 1.48; 95% CI, 1.27–1.60), again confirming the importance of rapid growth in the first year of life.^43^

Secular trends in growth were investigated using waist circumference measurements taken in the CIF 4 times between age 2.5 years and age 5.0 years (from 1995 to 1998) compared with measurements taken in a cross-sectional sample of children of similar ages in 1987, up to 11 years earlier.^44^ BMI was also available for all of the children. Four age groups were compared with the sexes kept separate; boys waists were 0.60–1.99 cm larger in the mid-1990s than in 1987 (a 1.2%–4.1% difference), and girls waists were 1.34–2.50 cm larger (a 2.6%–5.2% difference; all *P *< 0.05). In both sets of children, waist circumference increased with age; however, in both sexes in 1987 and the mid-1990s, BMI declined between age 2 years and age 5 years—e.g., in boys in the ALSPAC, at age 2.5 years, the mean BMI was 16.7 (SD, 1.3) kg/m^2^ and at 5.0 years, it was 16.0 (SD, 1.4) kg/m^2^. This is consistent with the adiposity rebound occurring after age 5.0 years in the majority of children. At each age (except at age 5 y in boys), BMI was slightly higher in the mid-1990s than in 1987 (in boys, 1.4%–1.8% higher; in girls, 1.6%–4.1% higher; all *P *< 0.05).^44^ These data suggest that in the period between 1987 and 1995–1998 central fatness in children, measured by waist circumference, had increased and this increase was slightly faster than the concurrent increase in BMI.

##### Obesity development throughout childhood

The first published report that looked at obesity prevalence in the ALSPAC was from the CIF subsample at age 5 years.^45^ Higher frequency of obesity (7.2%) and overweight (18.7%) were found than expected when compared with the 95^th^ and 85^th^ percentiles of BMI, respectively, using the UK 1990 growth reference data (both *P *> 0.001). The incidence of obesity over the time span 3–15 years was investigated using the CIF subsample from age 3 years to age 15 years (n = 549) and the whole cohort from age 7 years to age 15 years (n = 4283) with the same definition of obesity as above.^46^ The 4-year incidence of obesity was highest between age 7 years and age 11 years (6.7% in CIF; 5.0% in the whole cohort), with incidence slightly lower between age 3 years and age 7 years (5.1% in CIF; data for whole cohort not available) and very much lower between age 11 years and age 15 years (1.6% in CIF; 1.4% in the whole cohort). In the CIF substudy, the risk of the child being overweight or obese at age 15 years was 2.4 (95% CI, 1.8–3.1) times higher if the child was overweight or obese compared with a healthy weight at age 3 years, 4.6 (95% CI, 3.6–5.8) times higher for the same at age 7 years, and 9.3 (95% CI, 6.5–13.2) times higher for the same at age 11 years.^46^ There were similar results in the whole cohort. These results suggest a cumulative effect on obesity development over childhood.

A separate analysis (with the sexes separated) investigated whether a child who was overweight (BMI *z*-score, ≥1.04 but <1.64 using the UK 1990 growth reference) at age 7 years was likely to progress to being obese (BMI *z*-score, ≥1.64) at age 13 years (n = 5175).^47^ Children who were overweight at age 7 years were much more likely (38% and 30% in boys and girls, respectively) than normal-weight children (both 5%) to become obese by age 13 years (adjusted OR for boys, 20.5 [95% CI, 12.6–33.6]; for girls, 16.4 [95% CI, 10.0–27.0]). More than 68% of children who were obese at age 7 years remained so at age 13 years.^47^ Adjustment for SEB and parental obesity did not attenuate these relationships to a meaningful extent.

Fat mass indices from bioimpedance were used to define excess fatness (using internal standards) at age 7 years and age 11 years, and excess fatness was tracked over time (n = 6066) in comparison with BMI-derived overweight and obesity (defined using IOTF cut-offs).^48^^,^^110^ Table 8 shows a very high degree of tracking in the normal-weight and fatness categories, such that children who were of normal fatness or BMI at age 7 years were very unlikely to become very over-fat or obese by age 11 years. The higher fatness categories were less stable than the higher BMI categories (Table 8) and very similar in boys compared with girls.^48^ At age 7 years, 11% of children had a parent who was obese (prepregnancy), and these children were more likely to be overweight (21%) or obese (10%) at age 7 years compared with children of normal-weight parents (15% and 3%, respectively). Over time, BMI tracked more strongly than fat and lean indices and seemed to reflect tracking of fat and lean mass equally.

**Table 8:** Persistence of obesity, overweight, and normal weight (defined by International Obesity Task Force cut-offs) and fatness categories (measured by bioimpedence) in children from age 7 years until age 11 years

IOTF categories	All (n = 6066)	Boys (n = 3008)	Girls (n = 3058)
Of total obese at age 7 y (n = 180), percentage at age 11 y			
Still obese	75	82	70
Overweight	23	16	28
Normal weight	1.7	1.4	1.9
Of total overweight at age 7 y (n = 762), percentage at age 11 y			
Obese	16	23	11
Still overweight	63	60	66
Normal weight	21	17	24
Of total normal weight at age 7 y (n = 5214), percentage at age 11 y			
Obese	0.5	0.5	0.4
Overweight	11	11	11
Normal weight	89	89	88
Fatness categories			
Total	5933	2938	2995
Of total very over-fat^a^ at age 7 y (n = 270), percentage at age 11 y			
Still very over-fat	57	61	54
Over-fat	30	30	30
Normal fat	13	9.1	16
Of total over-fat^b^ at age 7 y (n = 582), percentage at age 11 y			
Very over-fat	12	16	9
Over-fat	33	32	34
Normal fat	55	52	57
Of total normal fat at age 7 y (n = 5081), percentage at age 11 y			
Very over-fat	1.0	1.2	0.7
Over-fat	5.6	5.5	5.6
Normal fat	94	93	94

Reproduced from Wright C, et al. (2010)^48^ with permission.

^a^ >95^th^ internal percentile for fat *z*-score.

^b^ 85^th^–95^th^ internal percentile for fat *z*-score.

Taken together, these studies suggest that adolescent obesity has its roots in early and mid-childhood and is well-established before the age of 11 years.

##### Risk factors for obesity

The risk factors for obesity were investigated in the whole cohort at age 7 years by multivariable analyses using binary logistic models.^49^ There was no significant difference in frequency of obesity between boys (9.2%) and girls (8.1%). In the fully adjusted models for risk of obesity (n = 5893 with complete data), which included maternal education, birth weight was positively linearly associated (OR, 1.05: 95% CI, 1.03–1.07; *P *< 0.001), as was smoking during pregnancy (OR for 1–9 cigarettes/d, 1.76; 95% CI, 1.21–2.52; *P *< 0.01), both parents obese prepregnancy (OR, 10.44; 95% CI, 5.11–21.32; *P *< 0.001), child watching television for >8 hours per week at age 3 years (OR, 1.55; 95% CI, 1.13–2.12; *P *< 0.01), and nighttime sleep of <10.5 hours at age 2.5 years (OR, 1.45; 95% CI, 1.10–1.89; *P *< 0.01). Breastfeeding and age at commencement of complementary feeding, although associated in the minimally adjusted model, were not robust to full adjustment. In the CIF substudy (n = 909), in fully adjusted analysis, rapid early growth (highest quartile of weight SDS at 8 months compared with the rest [OR, 3.13; 95% CI, 1.43–6.68; *P* = 0.004) and early adiposity rebound (very early rebound compared with later; OR, 15.00; 95% CI, 5.32–42.30; *P *< 0.001) were independently associated with the risk of obesity.^49^ These data confirm that early life is a critical time in the development of mid-childhood obesity and that maternal and childhood lifestyle factors also play a part. These results reiterate that parental obesity is a very important determinant of childhood obesity.

It is likely that genetic variation is driving some of the association of parental obesity with offspring obesity. In this regard, the investigation of the *FTO* gene has been a priority, and the ALSPAC data were used in a multicohort investigation of age-dependent associations between variations in the *FTO* gene and BMI from early infancy to age 13 years.^50^ BMI was modeled using the LMS method,^107^ and median curves showed that carriers of the minor alleles had a lower BMI in infancy, an earlier adiposity rebound, and a higher BMI in later childhood. The allele effects were additive in that BMI curves of children with one minor allele were in-between those with 2 and those with none. Longitudinal analysis confirmed the cross-sectional analysis. These results provide important new insights in the role of the *FTO* gene in obesity development.

##### Inequalities in growth and obesity development

There are socioeconomic disparities in the United Kingdom for childhood growth and obesity development, and the age at which these start to emerge has been investigated using ALSPAC data. Height trajectories from age 0 to age 10 years were modeled (n = 12 366) across 4 maternal education categories, with the high educational attainment category split into A-level and degree groups.^51^ There was a clear positive gradient in birth length by maternal education category, and the differences in height had widened only very slightly by 10 years of age; the mean difference between the lowest and highest maternal education category at 10 years was 1.4 cm for boys and 1.7 cm for girls and was in proportion to the birth length differences. Trajectories of ponderal index at ages 0–2 years and BMI at ages 2–10 years were also investigated using maternal educational attainment categories.^52^ There was little evidence of patterning of ponderal index, but differences in BMI trajectory between maternal education groups started to emerge at 4 years and widened with increasing age. By age 8 years, there was a clear gradient in girls; the higher the educational attainment of the mother, the lower the BMI trajectory. For boys at age 8 years, only those with mothers within the highest educational attainment differed from the other 3 groups. At age 10 years, the mean BMI difference between the highest and lowest maternal education categories was 0.89 kg/m^2^ for girls and 0.38 kg/m^2^ for boys.^52^

Inequalities in fat mass assessed by DXA at age 9 years and in blood pressure measured at age 10 years were also assessed using maternal education categories.^53^ There were differences between boys and girls in fat mass associations confirmed by the interaction term (*P* = 0.0052). The slope index, highest to lowest maternal educational attainment for the geometric mean of fat mass, adjusted for height (null = 1.0) was 1.21 (95% CI, 1.08–1.36) for boys and 1.34 (95% CI, 1.23–1.46) for girls. As found for BMI trajectories, the difference in fat mass for girls showed a gradient over all of the maternal education groups, whereas for boys only those with mothers with the highest educational attainment were different. There was some evidence of inequality in blood pressure in these children (higher systolic and diastolic blood pressure in children with mothers in the lowest compared with the highest educational attainment); however, this was partly mediated by adiposity.^53^ Taken together, these findings suggest that interventions to prevent obesity are particularly necessary in the children of less-educated mothers and should start early in childhood.

### Diet in relation to growth and obesity development

#### Timing of adiposity rebound

The association of diet with the timing of adiposity rebound, when BMI starts to rise after a nadir in early childhood, was investigated in the CIF substudy using BMI measurements up to age 5 years (n = 772 with dietary information).^54^ Children with very early rebound, at or before age 3.5 years (n = 53 [6.9%]), were compared with those with an early rebound between ages 4 and 5 years (n = 156 [20.0%]) and those with a rebound after age 5 years (n = 563). There was no difference in BMI z-scores between the groups at age 3.5 years, but at age 4 years and age 5 years, those with very early rebound had much higher mean BMI z-score than those in the other groups (*P *< 0.001). For children with very early adiposity rebound, a higher proportion (23.3%) had at least 1 obese parent (prepregnancy) compared with children with early (16.4%) or later adiposity rebound (10.4%; *P *< 0.01 and *P *< 0.05, respectively). Timing of adiposity rebound was not independently related to maternal education. There were no dietary differences between the groups at either age 8 months or age 1.5 years, even after adjustment for body weight.^54^ However, although food records were collected at age 3.5 years and age 5 years, these have not yet been used to compare between adiposity rebound groups, and because the early rebound occurs after age 3.5 years and before age 5 years, it is likely that investigating diet at these ages would be much more informative.

#### Energy intake during childhood

The association between parental BMI and offspring BMI may have a dietary component as well as the known genetic components. An analysis of diet trajectory using data from the FFQ and diet diaries between age 3 years and age 13 years and its association with maternal prepregnancy BMI as a determinant and with child BMI at age 15 years as the outcome (n = 4197) was performed.^19^ Greater maternal prepregnancy BMI was associated with greater child EI at age 3 years; mean predicted EI increased by 4 kcal (95% CI, 3–5) per unit increase in maternal BMI (*P *< 0.001) after adjustment for maternal age, education, parity, and social class. EI between age 3 years and age 7 years was increased by 1 (95% CI, 0–1) kcal/year per unit increase in maternal BMI (*P *< 0.001). There was a slower rate of increase between age 7 years and age 13 years with an increment of −1 (95% CI, −1 to 0) kcal/year per unit increase in maternal BMI (*P* = 0.04). Predicted EI at age 3 years and change in EI during childhood was associated with child’s BMI at age 15 years.^19^ For every 10-kcal increase in EI at age 3 years, there was a 0.19 (95% CI, 0.17–0.20) kg/m^2^ increase in BMI at age 15 years (*P *< 0.001). After additional adjustment for previous EI, for every 10-kcal/year increase in EI between age 3 years and age 7 years, there was an increase in BMI at age 15 years (0.46; 95% CI, 0.35–0.57 kg/m^2^; *P *< 0.001). However, there was no association between change in EI at ages 7–13 years and BMI at age 15 years in the fully adjusted model (−0.13; 95% CI, −0.91 to 0.67 kg/m^2^; *P* = 0.75). The total adjusted association between maternal prepregnancy BMI and child BMI at age 15 years was 0.33 (95% CI, 0.30–0.36) kg/m^2^ per unit increase in maternal BMI (*P *< 0.001), and 18% (95% CI, 16.7%–19.4%) of this was accounted for by EI from age 3 years to age 13 years (*P *< 0.002).^19^

These results imply that the strong association shown in the ALSPAC between maternal BMI and offspring obesity is partially mediated by early and mid-childhood diet. For example, this suggests that an average 3-year-old child would consume approximately 40 kcal/day more if their mother had a BMI of 30 kg/m^2^ than if she had a BMI of 20 kg/m^2^; this is equivalent to 2–3 sweets/chocolates or 1 sweet biscuit a day.

#### Dietary energy density and obesity

It is possible that eating a highly energy-dense diet may predispose to increasing fatness over time. This was investigated in the CIF subsample by characterizing dietary energy density (without drinks) at age 5 years and age 7 years and looking at fat mass determined by DXA at age 9 years as the outcome.^26^ This timeframe covers, in part, the period (ages 7–11 y) when the incidence of obesity is at its highest in the ALSPAC.^46^ Children with excess adiposity at age 9 years were defined as those in the top 20% of the log FMI distribution, and their dietary intakes were compared with those of the rest of the children. There was no evidence of an association between dietary energy density at age 5 years and excess adiposity at age 9 years in minimally or fully adjusted models (OR, 1.12; 95% CI, 0.90–1.40; n = 459). However, there was a positive association between energy density at age 7 years and excess adiposity at age 9 years in a minimally adjusted model (OR, 1.18; 95% CI, 1.04–1.34), and this was strengthened in the fully adjusted model, particularly by adjusting for misreporting status (OR, 1.36; 95% CI, 1.09–1.69; n = 584). Other confounders included in the fully adjusted models were sex, total EI, energy from drinks, fat intake, fiber intake, maternal BMI (prepregnancy) and education, overweight status of the child at baseline (by BMI), and TV watching at age 4.5 years (hours per day by questionnaire).^26^

**Figure 1:**
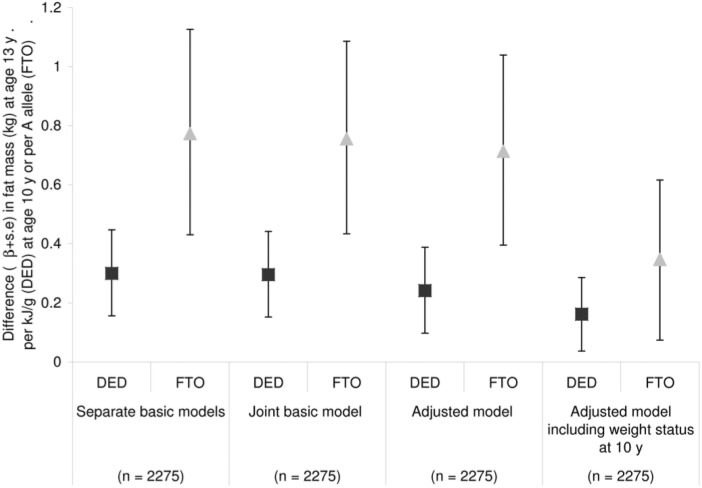
Predicting fat mass at age 13 years from dietary energy density (DED) at 10 years and *FTO* genotype in children (n = 2275). Reproduced from Johnson et al. (2009)^27^ with permission. Values are regression coefficients and 95% CIs. A statistically significant effect is indicated by a 95% CI that does not include 0. Separate basic models contain either *FTO* or DED and are adjusted for height at age 13 years and sex; misreporting of EI is included for models with DED. Joint basic model contains *FTO* and DED and is adjusted for height at age 13 years, sex, and misreporting of energy intake. Adjusted model includes *FTO* and DED adjusted for height at age 13 years, sex, puberty (Tanner stages 1–5) at age 13 years, misreporting of EI (under-, plausible-, or over-reporter), EI from drinks (kJ/d) at age 10 years, maternal education (none, vocational, O level, A level, or degree), TV watching at age 8 years (<1/1–2/2 h/d), physical activity at age 11 years (counts per minute). Adjusted model including overweight status at age 10 years contains the same variables as adjusted model in addition to overweight status at age 10 years (defined by IOTF criteria). *Abbreviatons:* CI, confidence interval; EI, energy intake; IOTF, International Obesity Task Force.

Dietary energy density was available again at age 10 years, and body fatness was assessed by calculating FMI at age 13 years from DXA measurements; in this analysis, the relationship between fatness and *FTO* genotype was also investigated.^27^ There was no evidence of a relationship between energy density in the diet at age 10 years and fat mass at age 13 years when controlling for height and sex only, but after adjustment for misreporting of EI, an association emerged (0.21kg; 95% CI, 0.12–0.30 increase in FMI for each kJ/g increase in dietary energy density; n = 5527). There was no evidence of an interaction between dietary energy density and the *FTO* gene in relation to FMI. Regression analysis with both energy density and *FTO* in the model confirmed their independent relationship with change in FMI (Figure 1) (n = 4318). The addition of weight status at age 10 years to the model attenuated both effect sizes but did not abolish the relationships (Figure 1). For energy density, this suggests that overweight children are more likely to consume energy-dense diets, and this supports a causal role for dietary energy density in increasing fat mass over time. For *FTO*, the attenuation suggests that the effect of *FTO* genotype on fatness is cumulative over time.^27^

Dietary intakes were investigated directly in relation to *FTO* genotype at age 10 years in 3589 children with plausible EI to assess whether the *FTO* locus might affect appetite.^55^ There was an association of the minor allele with increased dietary energy and fat intake even after adjustment for current BMI: total fat consumption increased by 1.5 g/day (*P* = 0.02 for each allele), and total energy consumption increased by 25 kJ/day (*P* = 0.03 for each allele). This provides some evidence of a direct effect of the *FTO* locus on food intake, suggesting that its effect on fatness may be, at least partially, mediated by diet.

Fast foods tend to be energy dense and a cross-sectional investigation was carried out into whether adolescents who frequently ate in fast-food outlets had a higher BMI than those who did not.^56^ Structural equation models were used with data collected from the parent and child FFQs at age 13 years and weights and heights collected in the research clinic at age 13 years (n = 3620). An analysis of types of foods eaten showed that increased frequency of eating at fast-food outlets was associated with higher consumption of unhealthy foods at home (chips, burgers, pizzas, pies) (β = 0.29; *P *< 0.001) and lower consumption of healthy foods (vegetables and raw fruits) (β = −1.02; *P *< 0.001).^56^ An analysis of frequency of visits to fast-food outlets showed an association with higher BMI *z*-score (β = 0.08; *P *< 0.001). In general, eating frequently in fast-food outlets was a marker of eating a more energy-dense diet.

#### Drinks in relation to obesity

The relationship between the drinks consumed at age 5 years and age 7 years and FMI at age 9 years and increase in BMI from age 5 years to age 9 years was investigated using linear regression in the CIF substudy.^29^ The analysis focused on sugar-sweetened beverages, but these drinks accounted for only 15% of drinks consumed and only 3% of total EI; therefore, the investigation was widened to include milk, diet soft drinks, water, and fruit juice intakes. The prevalence of overweight, defined by IOTF BMI cut-offs, increased by 5% between age 5 years and age 9 years. There was no evidence of an association between sugar-sweetened beverage intakes at age 5 years (n = 521) or age 7 years (n = 682) and FMI at age 9 years. Intakes of diet soft drinks at both age 5 years and age 7 years were positively associated with FMI at age 9 years in unadjusted analysis; however, this association was removed by adjustment for current BMI at age 5 years and age 7 years. At both ages, diet soft drink consumption was correlated with current BMI,^29^ so it is possible that parents had provided this type of drink to their children in the hope of limiting weight gain. The intakes of sugar-sweetened beverages in the ALSPAC were very similar to those in the NDNS 1997,^92^ where they contributed 3% and 4% of energy in children aged 4–6 years and children aged 7–10 years, respectively, and in the NDNS, as in the ALSPAC, consumption of diet soft drinks was greater than consumption of sugar-sweetened beverages.

The relationship between milk intake at age 10 years and later weight and fat mass gain was investigated in a subset of the children who had recorded their diet for a full 3 days at both age 10 years and age 13 years and had data on physical activity measured by accelerometer at age 13 years (n = 2270).^57^ Two separate analyses were performed on drinks, 1 on total milk intake and the other on flavored milk intake. All dairy foods were then combined for a further analysis.

For total milk, boys consumed more than girls (254 g/d compared with 193 g/d, respectively; *P *< 0.001) at age 10 years.^57^ For both sexes, approximately two-thirds of this was reduced-fat milk. There was some evidence that milk intake was negatively associated with body fat (%) measured at age 11 years in the fully adjusted model (*P* = 0.03), but there was no association with body fat (%) measured at age 13 years. There was, therefore, no evidence from this study that milk intake plays a role in relation to adiposity.

Flavored milk was consumed by 380 (16.7%) of the children at age 10 years, and there was no difference between consumers and nonconsumers in the percentage of children who were overweight/obese at age 10 years or in maternal educational attainment or maternal prepregnancy BMI.^58^ Flavored milk consumers had a higher average intake of energy, fat, carbohydrate, protein, and calcium but lower intake of fiber compared with nonconsumers. This amounted to almost 150 kcal extra EI per day for flavored milk consumers, but there was no difference in the amounts of other foods eaten, including fruits and vegetables. Change in weight and body fat was measured between age 11 years and age 13 years. In children of normal weight at age 10 years, the change in weight and body fat was similar between flavored milk consumers and nonconsumers. However, in overweight/obese children there was some evidence that the expected decline in body fat (%) was less in consumers (mean decline, −1.27%; 95% CI, −2.32 to −0.22) than in nonconsumers (mean decline, −2.90%; 95% CI, −3.42 to −2.37; *P*=0.007). This difference was attenuated but not removed by adjustment for pubertal status, maternal characteristics, and other foods in the diet.^58^ These results provide some limited evidence that overweight/obese children may benefit from avoiding flavored milk drinks.

When all dairy foods were combined, there was no association with excess fat accumulation, and the trend was in the direction of a negative association, particularly with full-fat dairy food intake.^59^ The data do not suggest that the consumption of dairy foods, in the amounts consumed in the United Kingdom, is related to obesity development.

### Diet and growth in relation to biochemical outcomes

#### Iron status

Iron status was investigated in relation to food and nutrient intakes at 1.5 years in the CIF subsample (n = 796).^60^ Ferritin concentration (a measure of stored iron) was more sensitive to nutrient intakes (energy-adjusted) than hemoglobin; calcium intake was negatively related to ferritin (*r* = −0.22; *P *< 0.001), and iron, vitamin C, and fiber intakes were positively related (all *P *< 0.05). The only robust relationship of nutrients with hemoglobin was positive with vitamin C intake (*r* = 0.13; *P* = 0.001). For particular foods, there was a negative association between the amount of cows’ milk consumed and ferritin (*r* = −0.25; *P *< 0.001) and a positive association between fruit (*P* = 0.024) and vegetable consumption (*P* = 0.030) and hemoglobin.^60^ Associations between fat intake (% energy) quartiles and hemoglobin and ferritin concentrations were also investigated (n = 666).^22^ There were no associations with hemoglobin, but ferritin concentration fell as fat intake quartile increased; 4.7% of children in the lowest quartile had a very low blood ferritin concentration compared with 14.8% in the highest (chi-squared *P* = 0.002). Both iron and vitamin C intakes were lower and calcium intakes higher in the highest compared with the lowest fat intake quartiles (*P *< 0.001), and these differences may account for the lower ferritin concentration found.^60^ Because the strongest association between nutrient intake and ferritin at age 1.5 years was a negative one with calcium intake (*P *< 0.001), it is possible that this is due to the presence of calcium in the gut adversely affecting the absorption of iron from the diet.^114^ Cows’ milk is the main source of calcium in the diet at this age (61% of calcium), and the amount of cows’ milk consumed was negatively associated with ferritin (*P *< 0.001). In the top quartile of fat intake, half of the 1.5-year-olds were consuming >500 g/day (20 oz/d) of cows’ milk. These data suggest that limiting cows’ milk intake to a maximum of 500 g/day (20 oz/d) in toddlers is sensible.

#### Blood lipids

The relationships of birth weight, current size, and central obesity with blood cholesterol and triglyceride concentrations at age 2.5 years (n = 385) and age 3.5 years (n = 470) in white singletons were investigated.^61^ Lipids were measured in nonfasting blood samples; therefore, although associations with cholesterol are likely to be only marginally compromised, associations with triglyceride should be treated with caution. Total cholesterol and triglyceride fell and high-density lipoprotein (HDL) cholesterol rose between age 2.5 years and age 3.5 years (all *P *< 0.001); sex differences in HDL cholesterol were found at age 3.5 years, with boys having higher concentrations. Height was negatively associated with triglyceride and total cholesterol concentrations at age 2.5 years in both sexes (*P* ranging from 0.091 to 0.016), but at age 3.5 years the only association evident was negative with triglycerides in girls (*P* = 0.010).^61^ The only association between BMI and lipid concentrations was negative with low-density lipoprotein (LDL) cholesterol in both sexes at age 3.5 years. Regression associations with birth weight were controlled for current height and BMI, central obesity, and breastfeeding history, and the only independent association was negative with HDL cholesterol in boys at age 3.5 years (β = −0.093 [standard error, 0.03]; *P* = 0.007). Central obesity was assessed by the ratio of waist-to-arm circumference, and there were some strong independent associations with lipid concentrations, using regression models adjusted for current height and BMI, birth weight, and breastfeeding history. At both ages in boys, increasing central obesity was associated with increasing triglyceride concentration (both *P *< 0.01). There was a negative association of central obesity with HDL cholesterol in boys at both ages (*P* = 0.028 and 0.002, respectively) with a marginal negative association only at age 3.5 years in girls (*P* = 0.072).^61^ LDL cholesterol showed a marginal negative association with central obesity in boys (*P* = 0.053) and a stronger quadratic association in girls (*P *< 0.010) at age 2.5 years but no associations at age 3.5 years.^61^ These results do not support a relationship between birth weight and blood lipid profile but do suggest that a central distribution of fat may be adversely related to blood lipid concentrations.

Dietary associations with blood lipid concentrations at age 2.5 years were investigated in relation to intake at age 1.5 years.^62^ There were some relationships between diet and total cholesterol in boys (n = 214); associations with energy-adjusted nutrients were positive with total fat (correlation coefficient *r* = 0.209; *P* = 0.002), saturated fatty acids (*r* = 0.211; *P* = 0.002), and total sugar (*r* = 0.152; *P *= 0.026) and negative with carbohydrates (*r *= −0.172; *P *= 0.012). In regression analysis that included these dietary variables and current height, the only independent relationships with cholesterol were with height (*P *= 0.044) and saturated fat intake (*P *= 0.001) in boys. Slightly higher total cholesterol was associated with consumption of compared with abstinence from particular foods, e.g., biscuits (*P *= 0.011) and chocolates (*P *= 0.012). There were no relationships between diet and HDL cholesterol in boys, and relationships with LDL cholesterol were similar to but weaker than those with total cholesterol. In girls (n = 133), there was a positive association between EI and HDL cholesterol (*r *= 0.204; *P *= 0.018) but no association with total or LDL cholesterol.^62^ The positive relationship with energy was independent of other nutrients in the regression model (*P *= 0.020), with some evidence of a negative relationship with polyunsaturated fatty acids (*P *= 0.036) emerging.

Associations between dietary fat intake (% energy) quartiles at 1.5 and 3.5 years and blood lipid concentrations at 2.5 and 3.5 years were examined.^22^ There was some evidence that total cholesterol in boys (at 2.5 y) was higher, on average, in the top 2 compared to the bottom 2 quartiles of fat intake at 1.5 years (*P *= 0.006). The relationship in girls was inconsistent. There were no associations between fat intake quartiles at 3.5 years and total or HDL cholesterol at 3.5 years in either sex.^22^ Relationships between diet and blood lipid concentrations in these preschool children were inconsistent over time and differed between the sexes.

#### Insulin-like growth factor

There are several insulin-like growth factors (IGFs) that play a part in regulating childhood growth, and these have been associated positively with the risk of several cancers and negatively with the risk of coronary heart disease in later life.^63^ Circulating concentrations of these hormones may be related to dietary intakes and may be a mechanism by which diet affects later health outcomes. Associations of growth and diet with the IGF system in childhood before the start of puberty were investigated. IGF-I concentrations were measured in blood collected at age 5 years and age 7–8 years, and height and components of height (trunk and leg length) were available in the CIF substudy.^63^ There were >200 boys and >200 girls with data at each age; in age-adjusted cross-sectional analysis in both sexes and at both ages, current height was strongly positively associated with IGF-I (all *P *< 0.002, after adjustment for maternal education, housing tenure, current BMI, and birth weight), leg length was not more strongly associated than trunk length, and birth weight was negatively associated (*P *< 0.006). Growth in stature between age 5 years and age 7–8 years and between age 7–8 years and age 9–10 years in relation to IGF-I was also examined. There were strong associations between IGF-I and subsequent growth in height in both sexes at both ages (all *P *< 0.004), with slight attenuation on controlling for confounders. In boys, leg and trunk length were equally associated; in girls, only trunk length was associated.^63^ These results do not support the hypothesis that associations between leg length and adult cancer risk are mediated through childhood IGF-I; it could be that age at onset of puberty (which is earlier in girls) confounds this relationship.

Dietary associations with IGF-I and IGFBP-3 (the main binding protein for IGF-I) were investigated in 521 white singletons at age 7–8 years in the ALSPAC. Intakes of milk and dairy products had been associated with IGF concentrations in adults previously, and because children are high consumers of these foods, they were likely candidates to examine.^64^ There was evidence of a sex difference in the relationship between milk or dairy product intakes and IGF concentrations; in boys only, both IGF-I and IGFBP-3 were higher in the top compared with the bottom quartile of intake of milk or dairy products. Adjusted analysis suggested that animal protein intake rather than milk or dairy per se might be the important factor.^64^ A separate analysis investigated the direct relationship between milk or dairy intake and height (*z*-scores) in these children (n = 744); again, there were statistically supported sex differences (e.g., interaction for sex × dairy products for leg length *P *= 0.015), with associations occurring only in boys.^63^ The association with dairy products was stronger with leg than trunk length. In multivariate analysis, adjustment for IGF-I attenuated the association to the null, suggesting that IGF-I mediates this association.

A further analysis examined other aspects of diet in relation to IGF concentrations.^65^ An age- and sex-adjusted analysis confirmed that protein, and in particular animal protein, was strongly positively associated with IGF-I (*P *< 0.001) and also with the ratio of IGF-I to IGFBP-3, which is a crude indicator of IGF-I bioavailability (*P *= 0.003). Positive associations for zinc, phosphorus, magnesium, calcium, and potassium with IGF-I were also found (all *P *< 0.008). However, in a regression model including protein and the 5 minerals listed above, only the association with protein was robust to adjustment (*P *= 0.020).^65^ There were negative associations between intakes of total fat, monounsaturated fatty acid, and polyunsaturated fatty acid and IGF-I (all *P *< 0.03), which were stronger in girls than in boys. IGFBP-3 was positively associated with EI (*P *= 0.002); other associations were much weaker and differed by sex. There were no associations with intakes of particular food groups, including meat, processed meat, and vegetables. Excluding under-reporters from the analysis did not change these associations; however, a positive association between EI and IGF-I emerged (fully adjusted *P *= 0.030).^65^ These 2 studies found important positive associations between animal protein intake and IGF-I; this was particularly important for dairy protein, which provides 24% of the total protein intake in these children. These results are consistent with observations in adults. There was also some evidence of a negative relationship between fat intake and IGF-I, which is consistent with the association of high fat and low IGF-I with the risk of coronary heart disease in adults.

#### Insulin

Low birth weight, rapid weight gain in the early years, shorter adult stature, and lower IGF-I concentrations have been shown to be associated with the risk of developing type 2 diabetes in adulthood.^115^ The opportunity was taken to investigate whether similar associations with growth parameters were present in ALSPAC children at age 7–8 years (n = 851) by using fasting insulin concentration and insulin secretion (30 min after an oral glucose load had been ingested) as a proxy for diabetes risk.^66^ Insulin and glucose concentrations (fasting and at 30 min) were used to calculate insulin sensitivity (a measure of how sensitive the body is to the effects of insulin) and insulin secretion (the amount of insulin produced in response to a glucose load). Girls had lower insulin sensitivity than boys (*P *< 0.0005), and this was not explained by body size. Insulin sensitivity decreased with increasing current weight, waist circumference, and BMI (all *P *< 0.0005). Height was inversely related to insulin secretion (*P *< 0.0005). Early rapid weight gain was associated with lower insulin sensitivity and elevated BMI at 8 years.^66^ Current BMI accounted for 10.2% of the variation in insulin sensitivity, and early weight gain accounted for 2.1%. There was no association between insulin sensitivity and birth weight except in children in the highest tertile for current BMI, whereby an inverse association was found (*P *= 0.0006). Insulin secretion correlated negatively with insulin sensitivity and was higher with elevated BMI and with rapid early weight gain (all *P *< 0.001).^66^ There were independent positive relationships of ponderal index at birth (adjusted *P *= 0.01) and childhood height (adjusted *P *= 0.047) with insulin secretion; thus, thinness at birth and smaller stature were related to reduced insulin secretion. IGF-I at 5 years (n = 252) predicted height gain between age 5 years and age 8 years (*P *= 0.008) and was positively related to insulin secretion at age 8 years (*P *< 0.001); this association was independent of current BMI (*P *= 0.004). The results are in line with findings in adult studies. The mechanisms for these relationships may be related to β-cell function and need further investigation, as does the contribution of diet.

#### Free fatty acids

The possibility that high concentrations of free fatty acids in the blood of the ALSPAC children may be associated with poor β-cell function, which could have manifested as reduced insulin secretion,^67^ was investigated. This was done in parallel with an adult study in which similar data were available. Higher fasting free fatty acid concentrations were associated with lower insulin secretion in both sexes in both groups (boys: *P *= 0.03; girls: *P *= 0.001; men: *P *= 0.03; women: *P *= 0.04). In the adults studied, higher fasting free fatty acid concentrations were associated with greater risk of developing type 2 diabetes in the following 5–8 years. Again, dietary intakes may contribute to these relationships, and the ALSPAC has the potential to investigate this in the future.

### Diet and growth in relation to other childhood outcomes

#### Birth weight associations with childhood diet

Low birth weight is associated with cardiovascular disease in later life. It is possible that birth weight might be related to differences in childhood diet, and these differences might mediate the association.^68^ The diet in the CIF substudy was investigated at age 8 months, 1.5 years, 3.5 years, and 7 years, and there was only minimal evidence of an association between a child’s birth weight and their later dietary intake; a small inverse association with saturated fat intake only at age 3.5 years was abolished in adjusted models. This suggests it is unlikely that diet plays a mediating role in the relationship between birth weight and later disease, at least in this cohort.

#### Puberty

Early onset of menarche in girls was associated with fast early growth; menarche age <12 years was associated with faster weight gain from age 0 to age 2 months (*P *= 0.006) and age 2 months to age 9 months (*P *< 0.001) but not with weight gain after that.^43^ Associations with length gain started after age 2 months and persisted to age 19 months. In logistic regression analysis, each 1 SD increase in weight SDS between age 0 and age 9 months was associated with a 34% increased risk for menarche at age <12.0 years (OR, 1.34; 95% CI, 1.21–1.49).

The relationship between dietary intake at age 3 years and age 7 years (by FFQ) and age 10 years (by food record) and age at menarche was investigated.^69^ The association found with EI at age 10 years was not robust to adjustment for current body size; however, relationships with protein intake at both age 3 years and age 7 years did persist (OR for reaching menarche by 12 y 8 mo per 1 SD increase in energy-adjusted protein intake at 3 y, 1.11; 95% CI, 1.00–1.23; *P *= 0.057 and OR at 7 years, 1.14; 95% CI, 1.04–1.26; *P *= 0.007). Meat intake appeared to be driving the association with protein at both age 3 years and age 7 years. For example, an early menarche was more likely in girls with an intake of >12 portions of meat compared with <4 portions of meat per week at age 7 years (OR, 1.57; 95% CI, 1.03–2.37; *P *= 0.020).^69^ The analyses were adjusted for maternal education and smoking, maternal age at menarche, parity, duration of breast feeding, and birth weight. These results suggest that high protein intake in early and mid-childhood may affect the timing of puberty.

The relationship between body composition at age 11 years and pubertal stage at age 12 years was investigated in boys and girls using BMI *z*-scores (UK 1990 reference standards) and bioimpedance-derived lean and fat indices (internal standardization).^48^ For girls, early pubertal development (breast development and pubic hair) was associated with higher mean BMI, fat, and lean mass index z-scores (BMI, 0.70 [95% CI, 0.59–0.79]; FMI, 0.23 [95% CI, 0.12–0.33]; lean mass index, 0.35 [95% CI, 0.26–0.45]). For boys, the association of early pubertal development (genitalia and pubic hair) with BMI z-score was variable, and lean mass index z-score showed a tendency to be higher (*P *< 0.05), whereas FMI z-score showed a tendency to be lower (*P *< 0.01).^48^ Thus, it is important to study boys and girls separately, particularly at the time of puberty.

#### Tasting ability

Bitter tasting ability was assessed in 4178 10-year-old participants who also had genetic data available relating to the TAS2R38 locus, which had previously been shown to be associated with bitter tasting ability.^70^ A taste score was measured and found to be lower in the group predicted to be nontasters by their genetic haplotype (median score, 3.7; interquartile range, 5.2) compared with the group predicted to be tasters (median score, 8.1; interquartile range, 2.5). A further analysis investigated how bitter tasting ability might relate to childhood growth and feeding behaviors.^71^ It is possible that children who are tasters might be choosier about what they eat than nontasters. Mothers had been asked about whether their child was choosy with food at several ages from 15 months to 4.5 years. More than half of the children were considered choosy by their mothers at 15 months, and the proportion increased with age. There was no evidence of a difference between tasting groups even at age 4.5 years when 78% of nontasters compared with 82% of “super” tasters were said to be choosy (*P *= 0.10). It may be that if a more strict definition of “being choosy” had been used, a significant difference would have been found. There were no differences in BMI at age 10 years between tasting groups but slightly more of the super tasters than the nontasters were below the 10^th^ percentile for height at age 10 years (12% compared with 8%, respectively; *P *= 0.008).^71^ These data warrant further exploration.

#### Autism spectrum disorders

The feeding behaviors, diet, and growth of children with autism spectrum disorders (ASDs) were investigated using the FFQs up to age 4 years.^72^ There were 79 children with ASDs, and they were compared with 12 901 control subjects. Children with ASDs started solid foods slightly later than control subjects and were more likely to be described as slow feeders at age 6 months. From age 15 months to age 4 years, they were consistently more likely to be described as difficult to feed (pooled OR, 2.92; 95% CI, 2.08–4.09; *P *< 0.001) and very choosy about food (pooled OR, 2.55; 95% CI, 1.91–3.40; *P *< 0.001). Nutrient intakes estimated from the FFQ at age 3 years did not differ between children with ASDs and control subjects, except those with ASDs had slightly lower intakes of vitamin C and ate less variety of foods, particularly consuming fruit and vegetables less often. There were no differences between children with ASDs and control subjects in weight, height, or BMI at age 18 months or age 7 years.^72^ These findings on eating behaviors are consistent with the idea that children with ASDs have difficulty accepting change.

## DISCUSSION

This review illustrates the great advantage that the ALSPAC has over cross-sectional studies of dietary intake and childhood growth because it follows the same children through childhood to adolescence with regular dietary assessments and measurements of size. The ALSPAC is able to identify when important changes in diet and growth occur. These valuable insights can inform decisions about the targeting of interventions to improve diet and encourage optimal growth, thus gaining the most efficient use of scarce resources. The contribution of longitudinal birth cohorts to scientific knowledge is now widely recognized.^116^ The ALSPAC has been in the vanguard of this type of research and has shown that including some measurements not immediately driven by a particular hypotheses can lead to important findings, e.g., those relating to iron status or autism. For each section of the results, the value added by assessing the publications from the ALSPAC in one review are discussed with the key findings, in brief, listed in Box 1.

Box 1Key findings in brief

- Diets in childhood changed decisively in the preschool years toward greater energy density. Increased intake of free sugars was the main reason for the greater energy density of the diet.
- Low intakes of vitamin D were common throughout childhood. At preschool ages, all ALSPAC children had dietary intakes below the UK dietary recommendations. Uptake of vitamin D supplements was low, in line with national surveys and despite recommendations that all children aged 1–5 years should receive them.
- Fruit and vegetable intakes were low at all ages; both family and child characteristics were important in determining intake.
- Maternal educational attainment was related to the quality of the child’s diet throughout childhood; lower education was associated with a more energy-dense diet with less fruit and vegetables consumed.
- There were 2 periods during which faster-than-average weight gain predisposed to obesity: early infancy and between age 7 years and age 11 years, when most children experience the adiposity rebound.
- Having 1 or both parents who were obese prior to pregnancy was one of the strongest risk factors for being obese in childhood. Genetic and dietary factors had associations that were independent of each other.
- Children who were overweight or obese in early or mid-childhood were much more likely than normal-weight children to be obese as adolescents.
- There were associations between the energy density of the diet in mid-childhood and the development of obesity at the time of the adiposity rebound.
- Levels of fatness in girls increased stepwise as maternal educational attainment decreased, but in boys, only those whose mothers had the highest educational attainment were slimmer than the rest.
- Interventions to prevent obesity are particularly necessary in the children of less-educated mothers; they should start at preschool age and include dietary change.
- Interventions to improve diet quality should aim to decrease intakes of sugary, energy-dense foods and increase intakes of fruit and vegetables. Parental involvement is key to success in improving children’s diets.

The longitudinal comparison of the nutrient and food group intakes over childhood in the ALSPAC clearly show that the greatest change in dietary intake occurs during the preschool period^15^^,^^16^ and that the diet remains relatively stable after that (Table 4).^15–18^ There is a step change in free sugars intake at this time from approximately 12% to approximately 16% of the energy intake. This is within the context of the recommendation of a maximum intake of 10% of energy from free sugars^93^ and is also seen cross-sectionally in the NDNS 2008–2012.^102^ Contributions of protein to EI decline slightly at the same time, and total EI rises in line with the size of the child (Table 4).^15–18^ Throughout childhood, average fiber intake is at only 75% of the amount thought to be adequate (Table 4). The increase in intake of sugars is likely due to increased numbers of consumers of energy-dense, noncore foods such as sugar confectionery, sweetened breakfast cereals, puddings, and ice cream (Table 5).^15–18^^,^^23^ The proportion of consumers of these types of foods remain similar at each succeeding age of assessment. The contribution to energy intake of these foods rises by 11% during this preschool period (Table 6).^23^ These results suggest that preschool could be a key time to provide a healthy eating environment for children and to educate parents about diet.

Overall, the diets of children in the ALSPAC were adequate for most nutrients, with the notable exception of vitamin D. At age 1.5 years and age 3.5 years, all of the ALSPAC children were found to be below the United Kingdom dietary recommendations for vitamin D intake, and children who had low intakes at age 1.5 years were twice as likely to have low intakes at age 3.5 years.^24^ Although it has been recommended for 25 years or more that children aged <5 years should be given vitamin D supplements,^93^ only a minority in either the ALSPAC^24^ or the national surveys actually were supplemented.^101^ It is possible that a fortification program could be a good option to improve this situation. It was also evident that relative intakes of other important nutrients such as calcium and vitamin A declined in the preschool years,^23^ and this was due to the children starting to consume more energy-dense, nutrient-poor foods (Table 6).

Fruit and vegetable intakes were below recommendations at all ages in the ALSPAC children, and low intakes were established before starting school (Table 5). The in-depth investigation of determinants of fruit and vegetable intake at age 7 years ^28^ confirmed that boys ate less than girls and that both family and child characteristics were important in determining intake. The ALSPAC results add to the evidence that mothers act as role models and gatekeepers for their children regarding healthy eating by showing that children whose mothers ate more fruit and vegetables and had rules about serving fruit and vegetables daily had greater intakes.^28^ Other studies have also found that the diet of the mother is a key influence on the diet of the child.^117^^,^^118^ In the ALSPAC, the child’s own eating behaviors, particularly choosiness with food, affected intake, especially of vegetables. Further support is needed to educate mothers about healthy food choices for their children and to encourage parents to eat well themselves.

The need to offer advice and help to parents was manifest in the investigation of school meal quality.^33^ The packed lunches brought from home by the children were almost always inadequate compared with recommendations,^93^^,^^99^ and other meals eaten during the day did not fully compensate for this.^33^ The cooked meals provided by the school were slightly better nutritionally than the packed lunches. Food-based standards for primary school meals were introduced in the United Kingdom in 2006, and a systematic review of studies assessing meals taken by children in schools throughout the United Kingdom has found an improvement in the provided school dinners but no improvement in packed lunches.^119^ Uptake of school dinners has increased since the time of the ALSPAC assessment, but quality standards must be maintained to maximize the benefit.

Misreporting of food intake, particularly by adolescents, is very likely in dietary assessment and has been found in many studies.^120^ The ALSPAC dietary studies have confirmed that as children progress to adolescence, the level of likely under-reporting of EI increases (Table 4)^15^^,^^16^^,^^24^^,^^26^ and that overweight or obese individuals are more likely to under-report their intake than normal-weight individuals.^18^^,^^31^ It is important to consider when assessing relationships between diet and various outcomes that misreporting affects all aspects of the diet due to the high correlation of energy with all macronutrients and most micronutrients. Furthermore, the ALSPAC data showed there was a bias toward the under-reporting of particular types of foods, often noncore and energy-dense foods. It is very difficult to find dietary assessment methods that are applicable in a population survey that are not subject to misreporting, and the ALSPAC results endorse the necessity of using statistical methods to account for misreporting to avoid misleading conclusions.^26^^,^^27^

The various analyses carried out using ALSPAC data suggest that in large-scale studies BMI provides a useful method (relatively cheap and easy) of assessing body size^35^^,^^38^; however, its use for measuring obesity may be more problematic. The use of waist circumference to assess obesity did not appear to have any advantage over BMI.^38^ Using BMI to determine obesity and overweight identified different individuals depending on the growth reference data applied.^34^^,^^42^ For example, when IOTF cut-offs for obesity were used, false-positive rates differed by sex, but there was no difference by sex when using the 95^th^ percentile of the UK 1990 growth reference standards.^42^ Several analyses of ALSPAC data confirm the benefit to be gained from assessing fat and lean body mass when studying growth and obesity.^36^^,^^37^ As with BMI, the different methods used were shown to identify different individuals as having excess fat.^36^ Although leg-to-leg bioimpedance is a cheap and easy method, there are difficulties in finding a suitable equation to calculate fat mass from bioimpedance.^37^ Using a DXA scan may give a slightly more precise measurement of fat and lean mass,^36^ but the equipment is expensive and not very portable, making this method impractical for most field work. These reports from the ALSPAC emphasize the importance of considering the method and growth reference data used to assess obesity when comparing results across studies.

Several analyses using ALSPAC data have examined growth trajectories and confirm 2 periods of fast growth: early infancy and between age 7 years and age 11 years.^39^^,^^42^ The latter is the time of increasing BMI known as the adiposity rebound. Investigations relating weight and ponderal index at birth to growth found positive associations with BMI and both fat and lean mass; however, ponderal index at birth was a better predictor of adiposity in adolescence than birth weight.^41^ Following from this, increases in ponderal index between age 0 and age 2 years and BMI between age 2 years and age 10 years were associated with greater fat mass at age 15 years (Table 7).^51^ ALSPAC data has also substantiated a secular trend toward increasing BMI over time.^44^ Furthermore, these studies highlight differences in growth rate between boys and girls (Table 7).^44^^,^^51^

The development of obesity throughout childhood has been the theme of several investigations of ALSPAC data, which identified that the highest incidence of obesity (defined by BMI) was between age 7 years and age 11 years; it was slightly lower between age 3 years and age 7 years and very low during adolescence.^46^ BMI is a measure of both fat and lean mass and tends to track through childhood. ALSPAC data showed that fat mass varies more over time than BMI (Table 8). Children who were of normal BMI in mid-childhood were very unlikely to become obese by the start of adolescence, whereas those who were already overweight or obese were likely to remain so.^47^^,^^48^ These results suggest that interventions to reduce obesity in schools would only be cost effective if targeted at overweight or obese children. There were very similar findings and conclusions in a large, nationally representative study of children in the United States who were followed from age 5 years to age 14 years between 1998 and 2007.^121^

When risk factors for mid-childhood obesity were investigated, rapid early growth and early adiposity rebound were found to be important predictors.^49^ Early adiposity rebound occurred in 7% of children at around age 3.5 years and in 20% between 4 and 5 years^54^ and was much more likely if either parent was obese (prepregnancy). Having one or both parents who were obese (prepregnancy) was a strong predictor for mid-childhood obesity.^49^ The genetic underpinning of this association was shown in investigation of the *FTO* genotype, where having the minor allele was associated with increasing BMI between age 7 years and age 11 years.^50^ On the diet side, greater maternal prepregnancy BMI was associated with greater child EI at age 3 years and rate of increase of EI up to age 7 years.^19^ Furthermore, the association of mothers’ BMI with offspring BMI continued into adolescence and was partially explained by the child’s EI in early childhood.^19^

The detailed dietary data collected in the ALSPAC during mid-childhood facilitated investigation of the association between the energy density of the diet and the development of obesity at the time of its highest incidence in these children.^46^ As children progressed into adolescence, diets with high energy density were increasingly associated with higher dietary EI.^28^^,^^63^ Greater energy density at age 7 years was associated with an increased risk of adiposity at age 9 years,^28^ with similar findings for energy density at age 10 years and fat mass at age 13 years (Figure 1).^63^ An independent association with fat mass at age 13 years was found for the *FTO* genotype of the child (Figure 1).^63^ These studies confirm that both diet and genetic make-up are important in obesity development.

Differences in diet associated with SEB of the family, particularly in relation to maternal educational attainment, were found; children of mothers with low education consume less fiber (nonstarch polysaccharide) and more free sugars with more noncore and fewer core foods, particularly fruit, than children of mothers with high education.^18^^,^^20^ It is probable that these dietary differences have a role to play in the difference in adiposity between children of mothers with different extents of educational attainment. Variations in childhood adiposity between education groups began to emerge by age 4 years.^51–53^ By late childhood, fatness in girls increased stepwise as maternal educational attainment decreased, but in boys, only those with mothers with the highest educational attainment were slimmer than the rest.^52^ Interventions to prevent obesity are particularly necessary in the children of the less-educated mothers; they should start early in childhood and include dietary change.

ALSPAC data were used to investigate relationships between diet and growth and biochemical markers of health. In the children at preschool ages, the relationship between diet and iron status was explored.^60^ Ferritin was negatively associated with calcium and cows’ milk intake and positively associated with iron intake, and hemoglobin was positively associated with vitamin C and fruit and vegetable intake. At each age of dietary assessment, many children, particularly girls, had low dietary iron intakes. Dietary advice should aim to maximize iron intake and absorption.

Further analyses examined associations between food and nutrient intakes and the IGF system and found, in particular, a positive relationship between IGF-I and animal protein intake.^64^^,^^65^ There were strong positive associations between IGF-I and growth in height.^63^ Diet-induced variations in the IGF axis in childhood may have implications for the long-term risk of several chronic diseases. Exploration of insulin profiles in relation to growth showed that high BMI in mid-childhood and rapid weight gain in infancy were associated with a less favorable insulin profile in mid-childhood.^66^ These findings were in line with adult studies and show the importance of monitoring childhood growth and exploring the relationship of faster-than-average growth in infancy and childhood with health indicators.

### Strengths and limitations

One of the major strengths of this study is the availability of detailed records of foods/drinks as consumed at many ages throughout childhood in a cohort of prospectively followed children. The ability to explore details of diet that this data provides is extremely valuable and cannot easily be matched by other studies. Further strengths are the careful collection of anthropometric data by trained staff, mostly annually, and the inclusion of more precise ways of measuring body composition as they became available and affordable—these include bioimpedance measures and DXA scan measures. These methods are able to assess fat and lean mass and have shown that fat mass is a more sensitive measure of obesity development in relation to dietary intake than BMI. The availability of prospectively collected confounding variables, as well as details of the pregnancy and infancy of the child, adds further value to this cohort. However, the timing of data collection has not always been ideal due to delays in obtaining ethical approval and/or funding. It has also been important to keep participant burden to a minimum.

There are several limitations that apply to all observational studies, and one is that causality cannot be proved. In some analyses, new statistical methods, such as Mendelian randomization, which uses knowledge of the genetic makeup of the cohort, has been used to help overcome this problem. Another inevitable consequence is incomplete data collection and follow-up when cohort members do not take part in all phases of the study or decide to drop out completely. The ALSPAC has made particular efforts over the years to retain the cohort and has been relatively successful^73^; however, only approximately half of the full cohort provided food records at age 7 years, and there was further attrition at later ages (Table 2). For some analyses, statistical methods have been used to account for missing data, and most have aimed to take account of the biases that may distort the results by adjusting for SEB. The ALSPAC is a geographically defined cohort, albeit reasonably representative of the population at recruitment; the results, therefore, need to be evaluated from this perspective. Members of the cohort were born in 1991–1992, and there have been many changes in life experience for children in the United Kingdom since that time; therefore, the results always need to be seen in this context. For growth and obesity development, there are reference data that can be used to set ALSPAC data in context. This is also true of dietary data because in the United Kingdom there is a rolling program of dietary assessment in nationally representative cross-sectional samples covering all age groups at least every 10 years. Thus, ALSPAC dietary data can be compared with dietary data collected at similar ages in similar and different eras so that changes in diet over time can be evaluated. The food and nutrient intakes of children at each age in ALSPAC have been compared with this data and shown to be very similar, suggesting that ALSPAC results may be generalizable in the United Kimgdom.

## CONCLUSION

The longitudinal assessment of diet in ALSPAC’s childhood cohort has identified a critical time of dietary change between the ages of 1.5 years and 3 years when diet moves decisively toward increased intake of free sugars and, thus, increased dietary energy density. Dietary energy density showed a strong association with increasing body fatness between the ages of 7 years and 9 years and between the ages of 9 years and 13 years once adjustments were made for misreporting of EI. These analyses suggest that the most promising area for dietary intervention in relation to obesity prevention is the manipulation of dietary energy density. The intervention should start during preschool and aim to support parents in continuing good dietary habits throughout childhood. The dietary changes should aim to reduce the energy density of the diet and include foods that have a high nutrient density. To this end, the foods to encourage are core foods such as fruits and vegetables of all types, plain potatoes, pasta and rice, high-fiber/low-sugar breakfast cereals, high-fiber bread, plain meat, and fish. Consumption of noncore foods, including sweets and chocolate confectionery, sweetened breakfast cereals, white bread, sweet biscuits, and fried potatoes, should be discouraged. It would be beneficial if the whole family were encouraged to eat in this way because mothers are influential as both role models and providers of food to children. It would be prudent to design food provision in preschools and schools to reinforce these healthier food habits and to work with the food industry to promote the recommended foods.
